# PGPB and/or AM Fungi Consortia Affect Tomato Native Rhizosphere Microbiota

**DOI:** 10.3390/microorganisms11081891

**Published:** 2023-07-26

**Authors:** Martina Nasuelli, Giorgia Novello, Elisa Gamalero, Nadia Massa, Susanna Gorrasi, Cristina Sudiro, Marie Hochart, Adriano Altissimo, Francesco Vuolo, Elisa Bona

**Affiliations:** 1 Dipartimento per lo Sviluppo Sostenibile e la Transizione Ecologica (DISSTE), Università del Piemonte Orientale, 13100 Vercelli, Italy; martina.nasuelli@uniupo.it; 2 Dipartimento di Scienze e Innovazione Tecnologica (DISIT), Università del Piemonte Orientale, 15121 Alessandria, Italy; elisa.gamalero@uniupo.it (E.G.); nadia.massa@uniupo.it (N.M.); 3 Dipartimento di Scienze Ecologiche e Biologiche, Università degli Studi della Tuscia, 01100 Viterbo, Italy; gorrasi@unitus.it; 4 Landlab S.r.l., 36050 Quinto Vicentino, Italy; c.sudiro@landlab.net (C.S.); m.hochart@landlab.net (M.H.); a.altissimo@landlab.net (A.A.); 5 Sacco S.r.l., 22071 Cadorago, Italy; f.vuolo@saccosrl.it; 6 Center on Autoimmune and Allergic Diseases (CAAD), Università del Piemonte Orientale, 28100 Novara, Italy

**Keywords:** soil microbiota, tomato, microbial biostimulant, plant growth promoting bacteria, rhizosphere, *Pseudomonas fluorescens*, *Bacillus amyloliquefaciens*, arbuscular mycorrhizal fungi

## Abstract

Tomatoes are one of the most important crops worldwide and also play a central role in the human diet. Microbial consortia are microorganism associations, often employed as bioinoculants, that can interact with the native rhizosphere microbiota. The aim of this study was to evaluate the impact of a bacterial-based biostimulant (*Pseudomonas fluorescens* and *Bacillus amyloliquefaciens)* (PSBA) in combination, or not, with a commercial inoculum Micomix (*Rhizoglomus irregulare*, *Funnelliformis mosseae*, *Funnelliformis caledonium*, *Bacillus licheniformis*, *Bacillus mucilaginosus*) (MYC) on the native rhizosphere communities and on tomato production. The trial was carried out using *Solanum lycopersicum* in an open field as follows: control full NPK (CFD), control reduced NPK (CRD), MYC, PSBA, PSBA + MYC. Bacterial population in the different samples were characterized using a next generation sequencing approach. The bioinocula effect on the native rhizosphere microbiota resulted in significant variation both in alpha and beta diversity and in a specific signature associated with the presence of biostimulants, especially in the presence of co-inoculation (PSBA + MYC). In particular, the high initial biodiversity shifts in the community composition occurred and consisted in the increase in the abundance of genera correlated to the soil acidification and in an enhanced density of nitrogen-fixing microbes. The results also highlighted the well-known rhizosphere effect.

## 1. Introduction

The commonly cultivated tomato (*Solanum lycopersicum* L.) is an annual crop originating from the Andes region in South America, and has gained a significant spot in the vegetable crop production worldwide. Second only to potatoes, the global production of tomatoes in 2021 was more than 189 million tons, distributed in 5 Mha, where 80% of this value is intended for industrial transformation [1,2]. Italy is the first European producer, with 6.6 million tons produced in almost 100 kha, mainly in the Southern Italy [2,3].

Besides the importance in the overall vegetable crop cultivation, tomatoes also have a central function in the human diet. They are rich in antioxidants [4] with beneficial properties from anticancer and immune boosting responses to bowel disease amelioration [1]. Considering the constant intensification of agricultural practices and artificial selection, tomato plants are more susceptible to soil borne diseases due to their genetic homogeneity [5,6], and the adverse effects caused by climate change are adding more threats to the tomato yields [3,7]. In this scenario, more sustainable but effective agricultural practices are needed, especially to ensure food supplies for a constantly growing population [8,9].

The soil microbiota harbors a multitude of organisms (e.g., viruses, archaea, bacteria, lower and higher eukaryotes) that are deeply influenced by both biotic and abiotic factors. Their interactions with plants’ radical apparatus have an effect on different agricultural aspects, such as plant overall health, yield production and fruit quality [8,10,11]. Considering their explicated positive impacts, microbial consortia, which are associations of microorganisms with non-conflicting relationships, are often employed as bioinoculants in a wide variety of crops. These inocula can interact with the native rhizosphere microbiota and influence the “rhizosphere feedback loop” [11]. Two main functional groups interact closely with crops showing their beneficial roles: plant growth promoting bacteria (PGPB) and arbuscular mycorrhizal fungi (AMF).

PGPB refer to a comprehensive set of bacterial genera that interrelate with crops, explicating both direct and indirect mechanisms that can boost the plant fitness by providing nutrients otherwise unavailable to roots, affecting the morphology and biomass and resistance to phytopathogens and abiotic stresses [5,11].

AMF are obligate symbiotic microorganisms belonging to the subphylum Glomeromycotina and are able to establish relationships with plants [12]. These fungi are able to produce an extraradical mycelium that improves the plant’s uptake of phosphorus and nitrogen [13]. Moreover, AM symbiosis can induce tolerance to various biotic and abiotic stresses [14,15] and modulate the secondary metabolism of the plant, also improving the plant fruit production and the fruit quality in terms of sugar and vitamin content [16,17].

Although the use of microbiota consortia has some limitations (i.e., interspecific competition, EU regulation on bioinocula, etc.), they are currently one of the research areas in sustainable agriculture with larger investments [18]. Furthermore, the possible bioinocula effect on the native microbiota community is well summarized in the review by Vuolo et al. [11] but is still poorly investigated, especially under real field conditions.

In tomato crops, soil bioinoculation has been reported to influence various aspects. Considering the PGPB inoculation, several bacterial strains have been shown to be involved in promoting tomato plant growth, improving yield and protecting against pathogens [19,20]. Zuluaga et al. [21] found that inoculation with different beneficial bacterial strains resulted in a shift in root metabolomics, with a consequent chemotaxis of beneficial bacteria leading to an improvement in tomato growth and biomass. Moreover, an initial strong microbiota has been shown to be essential for plant protection against pathogens with the presence of PGBP, such as *Pseudomonas* and *Bacillus* correlated with healthier plants [22], while different plant growth-promoting rhizobacteria have the ability to reprogram defense-related metabolism [23].

Regarding the beneficial effect of AM fungi in tomato roots, their presence is associated with a better uptake of substances, both spatially, through the formation of an extraradical micelium, and temporally, as AM fungi can guarantee a better nutritional status in the long term [24]. Furthermore, the presence of mycorrhizal fungi is associated with higher fruit quality, even when cultivated under conditions of nutrient scarcity [25] or high salinity [26]. Inoculation with AM fungi has also been correlated with a reduction in the severity of infection by pathogens such as *Phytophthora nicotianae* [14].

The effects of a mixed inoculation approach (PGPB + AMF) have been described by several authors as being more active to the single-kingdom inoculum. The application of bacterial and fungal biostimulants has been reported to reduce the incidence of *Fusarium oxysporum f.* sp. *lycopersici* (FOL) in tomato plants grown under controlled conditions compared to unmixed inocula [27]. The effects of mixed inocula are correlated with increased numbers, biomass and quality of tomato fruits, both under real agro-system conditions and under reduced fertilization conditions [28,29]. Furthermore, mixed inocula of fungi and phosphate-solubilising bacteria, combined with phosphorus-rich substrate, improved plant growth and yields while allowing for a reduction in fertilizer use and costs [30].

While many studies focus on the direct effects of soil bioinoculants on tomato crops, there is a gap on the combined effects of bioinoculants on plant productivity and simultaneously on the native rhizosphere microbiota, especially along the successional phenological phases. The aim of this study was to determine the impact of the addition of different types of bioinocula on the native resident microbiota in the tomato rhizosphere while subsequently describing the possible interactions that can be established between soil microorganisms and how they translate into positive effects for the plant, considering two different phenological states of tomato crop cultivation.

## 2. Materials and Methods

### 2.1. Field Trial: Experimental Setup and Plant Growth

The aim of the experiment was to assess the effects and the impact of different microbial and/or fungal inocula on tomatoes grown in open soil conditions and subjected to reduced fertilization. Therefore, the experimental plan included five treatments: (1) control full NPK (CFD): uninoculated plants fertilized according to conventional practice; (2) control reduced NPK (CRD): uninoculated plants; (3) MYC: plants inoculated with the a commercial inoculum Micomix (three AM fungi and Bacillus spp., Atlantica Agricola, Alicante, Spain), reduced NPK; (4) PSBA: plants inoculated with *Pseudomonas fluorescens* and *Bacillus amyloliquefaciens*, reduced NPK; (5) PSBA + MYC: plants inoculated with *P. fluorescens* and *B. amyloliquefaciens* and Micomix, reduced NPK. Bacterial inocula were provided by Sacco srl. while Micomix by Atlantica Agricola. Micomix, as declared by the producer, was composed by three species of mycorrhizae: *Rhizoglomus irregulare*, *Funnelliformis mosseae*, *Funnelliformis caledonium* equivalent to 12,500 propagules/g, rhizobacteria of the genus *Bacillus* spp.: *Bacillus licheniformis* and *Bacillus mucilaginosus*, equivalent to a concentration of 1 × 10^10^ UFC/g, 5.42% *w*/*w* of total nitrogen, 5.42% *w*/*w* of organic nitrogen, 0.93% *w*/*w* phosphorous pentoxide (P_2_O_5_) water soluble, 7.5% *w*/*w* of calcium oxide (CaO) water soluble, 51.09% *w*/*w* total organic matter, 6.48% *w*/*w* iron (Fe) chelated by EDTA, 6.48% *w*/*w* manganese (Mn) chelated by EDTA, 0.76% *w*/*w* boron (B) water soluble, 0.48% *w*/*w* zinc (Zn) chelated by EDTA, 0.24% *w*/*w* copper (Cu) chelated by EDTA, 0.24% *w*/*w* molybdenum (Mo) water soluble. Dose per application for horticultural crop: 1–2 kg /ha. Both inocula were applied following the producer’s instructions.

**Figure 1:**
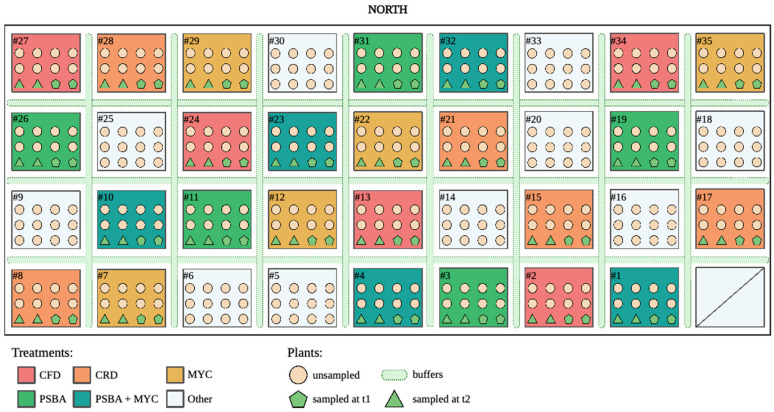
Experimental design. Randomized complete blocks were designed to test the 5 different formulated treatments, each consisting of 12 plants and tested in 5 replicates. A color scheme was assigned to each treatment and described in the legend: red for the uninoculated control plants with a full dose of NPK (CFD); orange for the uninoculated control plants with a reduced dose of NPK (CRD); yellow for plants inoculated with the commercial inoculum Micomix (MYC), reduced NPK; green for plants inoculated with *P. fluorescens* and *B. amyloliquefaciens* (PSBA), reduced NPK; dark green for plants inoculated with *P. fluorescens*, *B. amyloliquefaciens*, and the commercial inoculum Micomix (PSBA + MYC), reduced NPK. Circles indicate unsampled plants, while green symbols represent the tomato plants sampled at t1 (flowering phase) and t2 (fruit setting). Light green dotted lines belong to buffer plants used to separate the treated plots. Only reduced NPK fertigation was applied to these plants (Image of the plot created with BioRender.com).

The study design consisted of randomized complete blocks, as presented in Figure 1 (entries = 5; replicates = 5; plants = 5 × 5 × 12 = 300 + buffer plants between the plots, represented by light green lines).

The trial was carried out using *S. lycopersicum* var. Big Rio, a processing tomato variety, in an open field in Landlab (Landlab srl Società Benefit, Quinto Vicentino (VI), Italy, 45.57° N, 11.62° E, 33 m a.s.l.). A permeable mulch was used to decrease weed proliferation and reduce water loss by evaporation. Irrigation was performed by drip irrigation and was supplied according to plants’ demand. A hailstorm net was provided to protect the plants from possible adverse weather conditions.

Nutrients were provided by basal dressing and fertigation. A reduction in NPK supply was performed on treated entries, buffers and the “CRD, reduced NPK”. The products were applied at planting (0 days after planting (DAP)) and at 20 DAP.

Pest control was applied to the crop when pressure disease increased, according to the following plan: 24 June 2022: Pergado, Sivanto (Syngenta); 6 July 2022: Ridomil gold R (Syngenta); 15 July 2022: Matacar (Sipcam), Vertimec (Syngenta), Movento (Bayer); 22 July 2022: Aspor (Isagro spa); 29 July 2022: Oikos (Sipcam), Matacar (Sipcam); 5 August 2022: Aspor; 12 August 2022: Oikos, Sivanto, following the manufacturer’s instructions. All cultivation measures were performed in accordance with the conventional recommendations for the cultivar growing.

Conventional fertilization (CFD) consisted of a base dressing before transplanting, with N (45 kg/ha), P_2_O_5_ (70 kg/ha) and K_2_O (72 kg/ha) and for the entire growing period of N (105 kg/ha), K_2_O (168 kg/ha). Reduced fertilization (CRD and other treatments) consisted of a base dressing with N (31.5 kg/ha), P_2_O_5_ (49 kg/ha) and K_2_O (50.4 kg/ha) and for the entire growing period of N (105 kg/ha), K_2_O (168 kg/ha).

The air temperature and rain events were collected by means of a Landlab weather station (Figure S1).

The rhizosphere soil was sampled at different timepoints (t0: 10 samples to characterize the microbial community in the soil before the inoculum and transplant; t1: at the beginning of flowering; t2: at fruit setting) to evaluate its dynamics after the inoculation of microorganisms. Two plants per plot were used for sampling at timepoints t1 and t2 (Figure 1). Soil samples were maintained at −80 °C until the DNA extraction. Physical–chemical analyses were performed on each soil sample according to D.M. 13 September 1999.

At harvest time, tomatoes were collected and divided into two categories: marketable and unmarketable. Unmarketable fruits were further divided into green tomatoes and tomatoes affected by blossom-end rot (BER). Fruits were counted and weighed, and 10 marketable fruits per plot were analyzed for firmness and °Brix. All data were subjected to the analysis of variance (ANOVA) appropriate to the experimental design to evaluate the effects of treatments using XLSTAT software 2022.4.1 (Addinsoft, 2021, Paris, France). Comparison of treatment means was carried out using the Duncan test at significant level of *p* value < 0.05.

### 2.2. Microbial Community Characterization

The metagenomic workflow was carried out according to Bona et al. [31]. Briefly, total genomic DNA was extracted by DNeasy^®^ PowerSoil^®^ Kit (Qiagen, Hilden, Germany) using 0.25 g of soil following the manufacturer’s instructions. DNA of each sample was quantified by fluorimetric method according to the Qubit^®^ 2.0 Fluorimeter protocol.

Libraries were prepared by following Illumina 16S Metagenomic sequencing library preparation protocol in two amplification steps: an initial PCR amplification using *locus*-specific PCR primers and a subsequent amplification that integrates relevant flow-cell binding domains and unique indices (NexteraXT Index Kit, FC-131-1001/FC-131-1002). Primer sequences were: 16S-341F 5′-CCTACGGGNBGCASCAG-3′; 16S-805R 5′-GACTACNVGGGTATCTAATCC-3′, amplifying the hypervariable V3-V4 regions. Libraries were sequenced on NovaSeq instrument (Illumina, San Diego, CA, USA) using 250-bp paired-end mode.

### 2.3. Bioinformatic and Statistical Analyses

First, fastq file elaboration consisting of base calling, demultiplexing and adapter masking was performed with Illumina BCL Convert v3.9.31. Then, a clipping routine was applied to remove low-quality bases. Reads were then retained if they maintained a minimum length of 200 bp. Following the QIIME pipelines, the USEARCH algorithm (version 8.1.1756, 32-bit) allowed the following steps: chimera filtering, grouping of replicate sequences, sorting sequences per decreasing abundance and OTU identification. The operational taxonomic unit (OTU) picking aims to group query sequences into clusters, represented by centroids. Each centroid shares a level of similarity with its member sequences. An open-reference algorithm was used as a default approach unless differently enquired. All reads are used in the analysis if they maintain a minimum length of 200 bp after the removal of primer sequences and low-quality bases. Paired reads with permissive overlap at their 3 feet ends are merged into a single fragment and used as such to improve assignment accuracy. Reads that do not support overlap were maintained in the pool for downstream processing.

In an open reference analysis, sequences not matching any reference sequence of the database constitute a novel OTU and the most abundant and long read in each OTU was selected as the representative sequence. OTUs were built de novo with a clustering threshold set at 97%, with sequences that passed a pre-filter step for minimum identity of 90% with any sequence present in the reference database. OTUs in open reference analysis were generated with a minimum of 2 sequenced fragments. The RDP classifier and reference database were used to assign taxonomy with a minimum confidence threshold of 0.50. The Silva138 database was employed as 16S rDNA sequence reference.

After this elaboration, statistical analyses were performed using MicrobiomeAnalyst. First, data filtering was used to identify and remove features having low count and variance based on their abundance levels (minimum counts 30) across samples (prevalence). The statistical analysis regarding the biodiversity inside and between the different microbial populations was performed using “label” (e.g., CFD_t1; CFD_t2, etc.), “time” (t0, t1, t2) and “treatment” (CFD, CRD, etc.) as factors by considering: i) alpha diversity, characterized by the total number of observed species, Shannon and Simpson indexes, performed using the phyloseq package [32] and ii) beta diversity performed using the phyloseq package [32]. The distance between samples was measured using Bray–Curtis distance, and principal coordinate analysis (PCoA) was used to visualize the matrix in a 2D plot, where each dot represents the entire microbiome of a single sample. In this analysis, the statistical significance of the clustering pattern in ordination plots can be evaluated using permutational ANOVA (PERMANOVA).

Finally, the linear discriminant analysis effect size (LDA-LEfSe) method was applied at the genus level to identify the signature associated with the different parameters. This method is specifically designed for biomarker discovery and explanation in high-dimensional metagenomic data [33]. It incorporates statistical significance with biological consistency (effect size) estimation. It performs the non-parametric factorial Kruskal–Wallis (KW) rank-sum test to identify species with significant differential abundance with regard to the factor of interest, followed by linear discriminant analysis (LDA) to calculate the effect size of each differentially abundant feature. The result consists of all the genera with the highest mean and the logarithmic LDA score (effect size). Features are considered significant based on their adjusted *p*-value. The default *p*-value cutoff was 0.05.

### 2.4. Root AM Colonization Assessment

Mycorrhizal colonization was assessed starting from a sample of 40 randomly chosen 1 cm long root pieces for each plant. These root samples were cleared in 10% KOH for 45 min at 60 °C, stained with 1% methyl blue in lactic acid and mounted on a slide. Mycorrhizal colonization was estimated according to Trouvelot and coworkers [34]: frequency of mycorrhization (F%), mycorrhizal degree (M%), frequency of arbuscules (A%) and frequency of vesicles (V%) were calculated. Data were statistically analyzed by one-way ANOVA (using “label” as factor) followed by Tukey HSD *post hoc* test with Bonferroni adjusted *p*-values. A two-way ANOVA was also performed using “time” and “treatment” as factors. Differences were considered significant for *p*-values < 0.05.

## 3. Results

### 3.1. Soil Analysis

The physical–chemical analyses of the soil were performed on the samples collected at the baseline conditions, and the characteristics are reported in Table 1.

The soil texture is silty loam as reported by ARPAV (https://gaia.arpa.veneto.it/maps/293/view, 24 July 2023).

Considering the parameters of soil fertility, pH was found to be 8.29, classifying the soil as alkaline. The electrical conductivity (EC) was low. The active lime was in the reference ranges (1.93%), while the available phosphorus was reported as low (19.3).

The measure of organic matter and total nitrogen are in the reference ranges (1.69 and 1.038, respectively), while the C/N ratio was 9.44, slightly lower than to the standard values. Compared to the micronutrient elements, boron quantity resulted low (<0.50), while the values of iron, manganese, and copper were higher than the reference ranges. Only zinc presented values in the standard intervals.

For the exchange complexes, exchangeable sodium and potassium showed very low and low quantities, respectively, while ion calcium was listed as very high. Ion magnesium had a normal value of 2.39. Base saturation and CEC were, respectively, normal and very high compared to the reference ranges.

### 3.2. Production

The CRD treatment showed 60% lower production (Table 2), in terms of marketable fruits compared to CFD, although this difference is not statistically significant. However, if all the production is considered (marketable + green fruits), the difference is significant (Table 2). All the treatments performed better than the CRD (from +13.1% to +60.1%) and showed a lower percentage of BER fruits.

No statistically significant difference was found in quality parameters (firmness and °Brix) (Table 3).

### 3.3. Soil Microbiota Profiling

A total of 133,072,948 reads were obtained with a mean value of 1,073,169 reads per sample. The genomic sequences were included in the BioProject PRJNA916628, titled “Impact of PGPB bacteria and AM fungi inocula on resident communities associated with tomato roots”, available in NCBI database https://submit.ncbi.nlm.nih.gov/subs/sra/SUB12451409/overview, accessed on 20 December 2022. The BioProject contains 122 objects.

#### 3.3.1. Alpha Diversity

**Figure 2:**
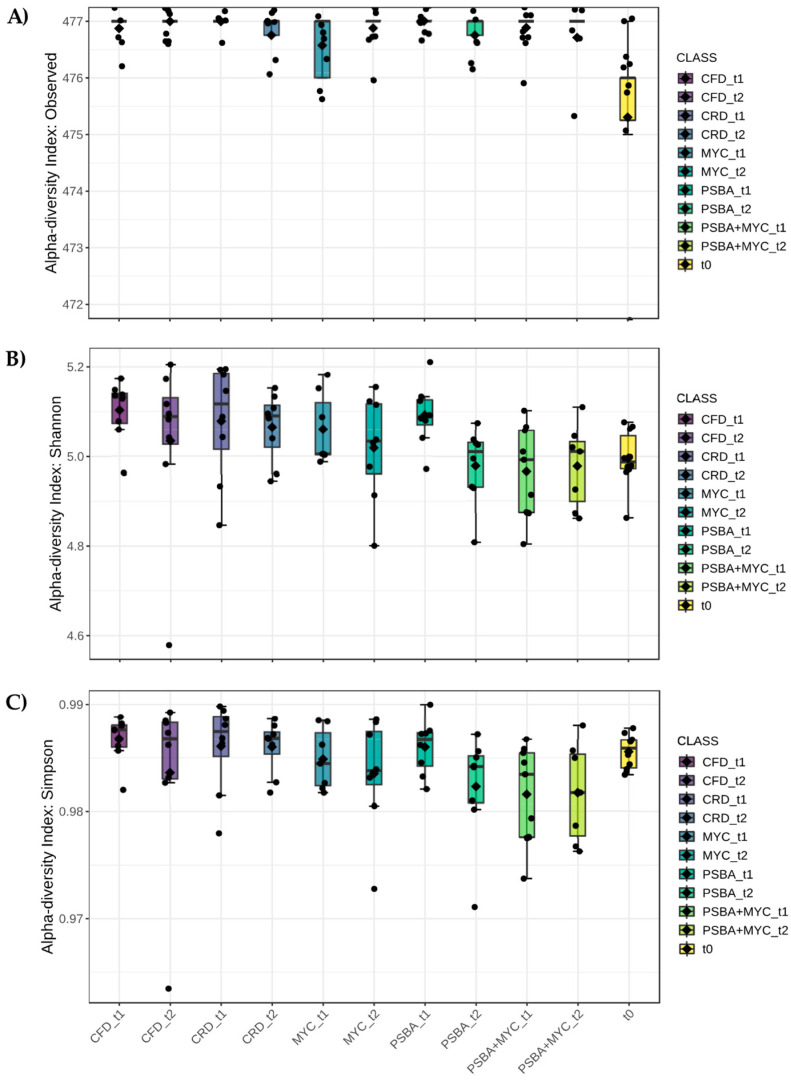
Alpha diversity analysis considering the label. (**A**) Number of observed species (*p*-value 0.000445), (**B**) Shannon index (*p*-value 0.0155) and (**C**) Simpson’s index (*p*-value 0.0706). *p*-value cut-off for significance is 0.05. In the figure, black diamond indicated the mean value while the black line inside the box represented the median value. Alpha diversity analysis was performed using the phyloseq package of MicrobiomeAnalyst. CFD—uninoculated control plants with a full dose of NPK; CRD—uninoculated control plants with a reduced dose of NPK; MYC—plants inoculated with the commercial inoculum Micomix, reduced NPK; PSBA—plants inoculated with *P. fluorescens* and *B. amyloliquefaciens*, reduced NPK; PSBA + MYC—plants inoculated with *P. fluorescens*, *B. amyloliquefaciens*, and the commercial inoculum Micomix, reduced NPK.

**Figure 3:**
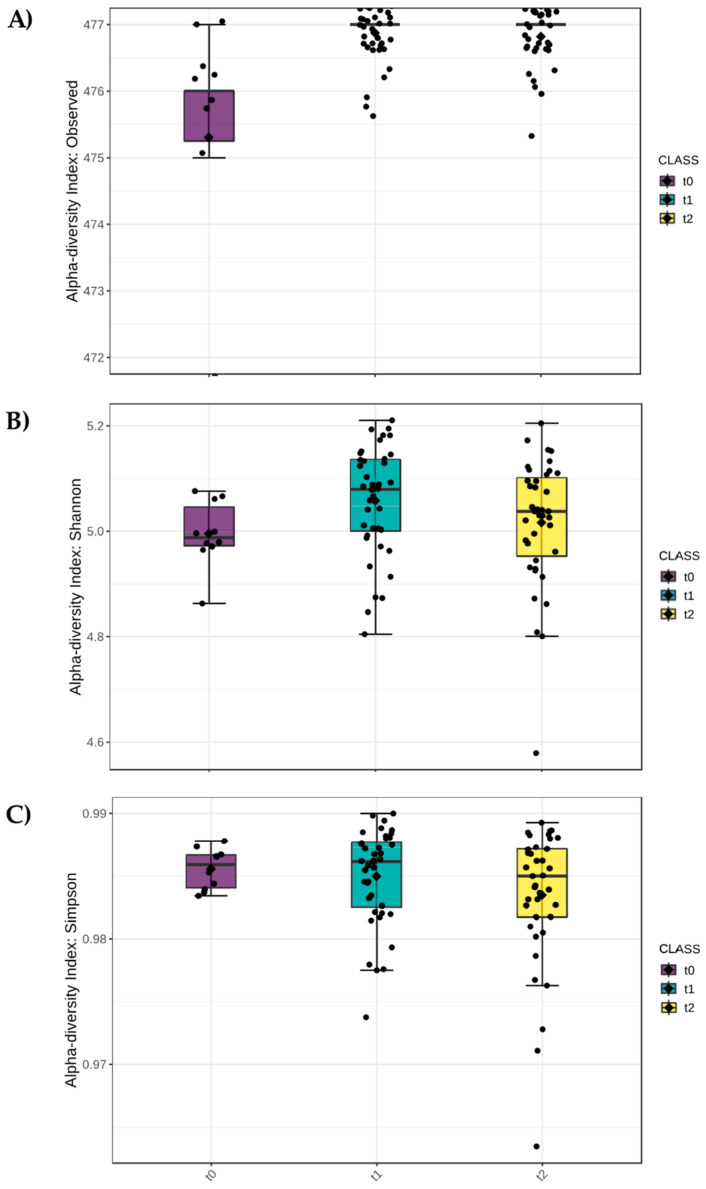
Alpha diversity analysis considering time as factor. (**A**) Number of observed species (*p*-value < 0.0001), (**B**) Shannon index (*p*-value 0.0564), and (**C**) Simpson’s index (*p*-value 0.4612). *p*-value cut-off for significance is 0.05. In the figure, black diamond indicated the mean value while the black line inside the box represented the median value. Alpha diversity analysis was performed using the phyloseq package of MicrobiomeAnalyst.

**Figure 4:**
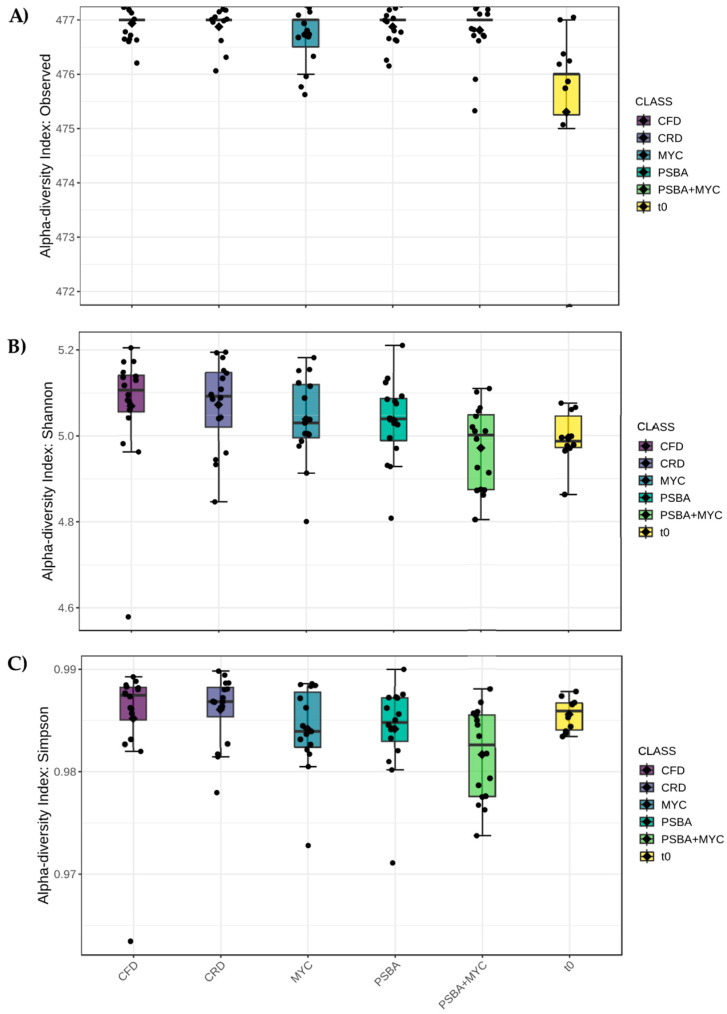
Alpha diversity analysis considering the treatment factor. (**A**) Number of observed species (*p*-value < 0.0001), (**B**) Shannon index (*p*-value 0.0088), and (**C**) Simpson’s index (*p*-value 0.0192). *p*-value cut-off for significance is 0.05. In the figure, black diamond indicated the mean value while the black line inside the box represented the median value. Alpha diversity analysis was performed using the phyloseq package of MicrobiomeAnalyst. CFD- uninoculated control plants with a full dose of NPK; CRD- uninoculated control plants with a reduced dose of NPK; MYC- plants inoculated with the commercial inoculum Micomix, reduced NPK; PSBA- plants inoculated with *P. fluorescens* and *B. amyloliquefaciens*, reduced NPK; PSBA + MYC- plants inoculated with *P. fluorescens*, *B. amyloliquefaciens*, and the the commercial inoculum Micomix, reduced NPK. t0- microbial diversity at baseline.

Alpha diversity was evaluated according to label (Figure 2), time (Figure 3) and treatment (Figure 4) factors using three different estimators: the number of observed species (A), Shannon (B), and Simpson (C) indexes.

Considering the label factor (each biological treatment at each time considered), all analyzed populations showed a higher number of observed species than at baseline (t0), i.e., soil-associated microbial populations in the absence of the tomato plants (Figure 2A). The Shannon index had very high values, ranging from 5.0 to 5.2 (Figure 2B), with significant differences compared to the different experimental conditions. The same trend was observed also for the Simpson index, but without significant differences with respect to the label factor (Figure 2C) with values near one, which is the maximum value that can be reached by this index.

Considering the time factor, populations at sampling times t1 (at the beginning of flowering) and t2 (at fruit setting) showed a higher number of observed species than at baseline (t0), with a high significant difference (*p*-value < 0.0001). Moreover, t1 and t2 populations were characterized by a comparable number of species (Figure 3A). Both Shannon (Figure 3B) and Simpson (Figure 3C) indexes had very high values without significant differences between times.

Considering the treatment factor, populations at sampling times t0 (at baseline) were confirmed to be the ones with the lower number of the observed species (Figure 4A). The populations showing the highest values for the Shannon and Simpson indices were those combined with the presence of uninoculated plants (CFD and CRD). Moreover, a decreasing trend of the biodiversity values in the presence of fungal (MYC) or bacterial (PSBA) inoculation, and even more in the combined inoculation (PSBA + MYC) treatment, was observed (Figure 4B,C).

#### 3.3.2. Beta Diversity

**Figure 5:**
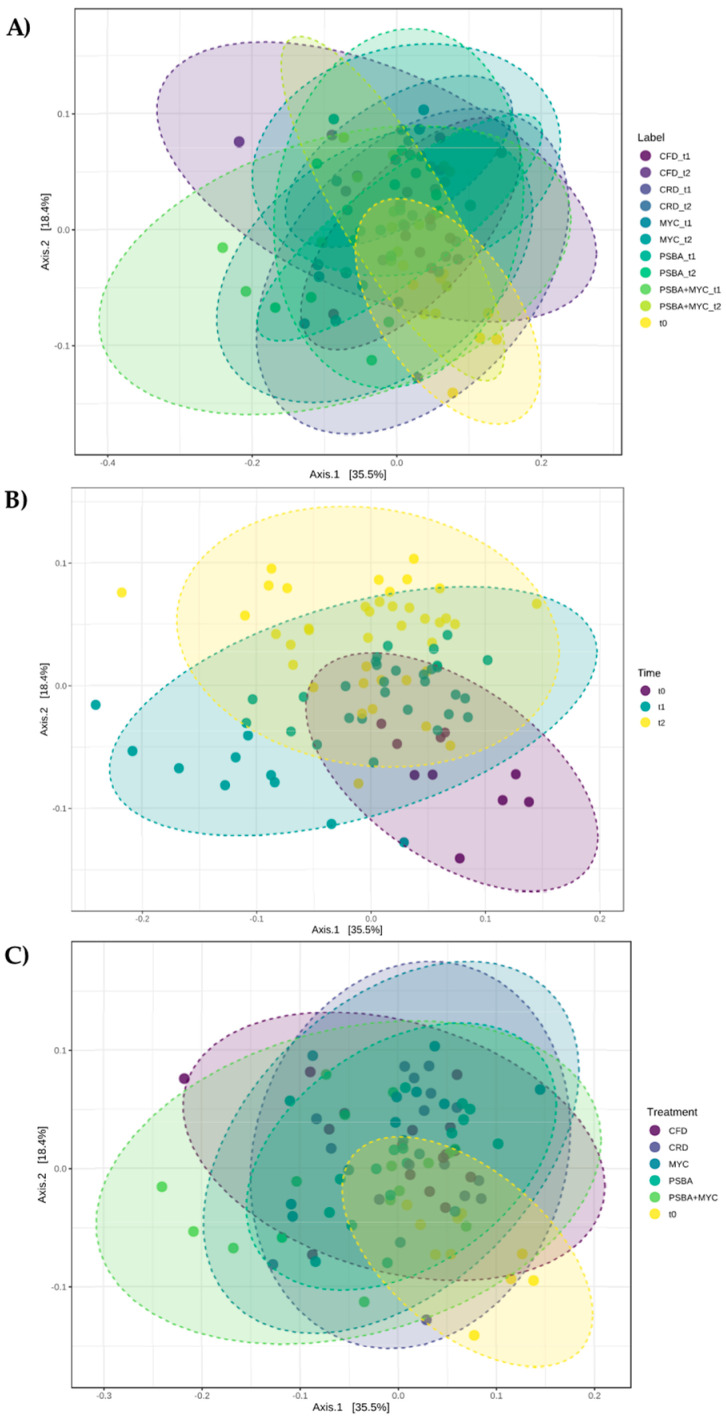
Beta diversity analysis at the genus level. Principal coordinate analysis (PCoA) based on Bray–Curtis metrics shows the dissimilarity of bacterial communities in the different soils according to (**A**) label (*p*-value < 0.001), (**B**) time (*p*-value < 0.001), (**C**) and treatment (*p*-value < 0.001). Beta diversity analysis was performed using Microbiome Analyst.

Beta diversity underlines the degree of distance between the different analyzed groups, giving a measure of comparison between bacterial communities. We estimated the beta diversity according to labels (Figure 5A), time (Figure 5B), and treatment (Figure 5C).

Principal coordinate analysis (PCoA) based on Bray–Curtis metrics revealed that populations were different according to label, time, and treatment factors, showing a high diversity in the genus members of the soil communities. As already highlighted in the alpha diversity analysis, the baseline microbiota showed a higher degree of dissimilarity compared to the other groups. However, a core microbiota is recognizable considering all the tested parameters.

#### 3.3.3. Signature

##### Signature Associated with the Presence of Tomato Plant

Data regarding the significant genera determining the variation in biodiversity associated with the presence of tomato plants are shown in Table 4. The table, comparing the treatments at each time to the baseline microbiota (t0), shows the genera (30%) that are significantly influenced by the plant. Positive differences greater than 10% are highlighted in green (54% of the significant genera), while negative differences greater than 10% are highlighted in red (25% of the significant genera). In particular, among the genera positively affected, we can find: *Acidibacter*, *Aeromicrobium*, *Allorhizobium*, *Neorhizobium*, *Pararhizobium*, *Rhizobium*, *Altererythrobacter*, *Arthrobacter*, *Blastocatella*, *Bosea*, *Bradyrhizobium*, *Caenimonas*, *Chthoniobacter*, *Devosia*, *Ensifer*, *Fimbriiglobus*, *Flavisolibacter*, *Flavitalea*, *Herpetosiphon*, *Leptolyngbya EcFYyyy00*, *Lysobacter*, *Mesorhizobium*, *Microbacterium*, *Nitrospira*, *Pontibacter*, *Pseudoxanthomonas*, *Ramlibacter*, *Rhizobacter*, *Rhodopirellula*, *Sphingobium*, *Streptomyces*, *Terrimonas*, and *Variovorax*. On the contrary, some of the genera negatively affected were: *Anaerolinea*, *Anaeromixobacter*, *Burkholderia*, *Caballeronia*, *Parburkolderia*, *Cohnella*, *Dongia*, *Fictibacillus*, *Gaiella*, *Herbinix*, *Kribbella*, *Luedemanella*, *Lutispora*, *Marmoricola*, *Mycobacterium*, *Nocardioides*, and *Solirubrobacter*.

Considering not only the presence of the tomato plant, but also the phenological state (flowering and fruit ripening, Table 4 and Table 5, no significant genera were associated with the phenological shift.

##### Signature Associated with the Biostimulant Treatment

The results of the significant genera determining the variation of the microbial communities associated with the different biostimulant treatments are presented in Table 6, Table 7, Table 8, Table 9 and Table 10. Comparing the microbial communities associated to the different biological treatments to CRD (without any bioinocula), it is possible to highlight that some genera were significantly enhanced: *Alsobacter*, *Aminobacter*, *Leptolyngbya* EcFYyyy00, *Luteitalea*, *Nakamurella*, *Rhodocytophaga*, *Skermanella*. On the other hand, some genera seemed to be reduced compared to the CRD treatment, such as *Acidibacter*, *Altererythrobacter*, *Bradyrhizobium*, *Chloroflexus*, *Cupriavidus*, *Devosia*, *Fictibacillus*, *Hirschia*, *Ideonella*, *Luteolibacter*, *Mesorhizobium*, *Ohtaekwangia*, *Promicromonospora*, *Pseudoflavitalea*, *Pseudoxanthomonas*, *Sorangium*, *Sphingopyxis*, *Streptomyces*, and *Tahibacter*.

### 3.4. Mycorrhization Degree

**Figure 6:**
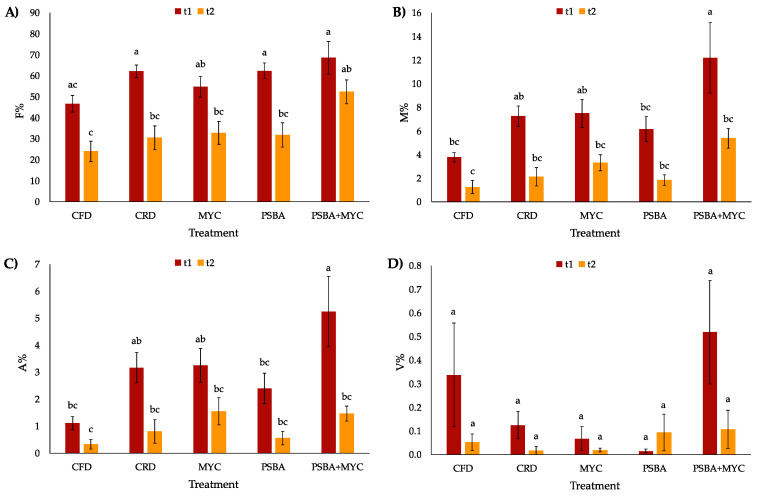
Mycorrhizal colonization in tomato plants. Different letters in the same color bars indicate significantly different values (*p* < 0.05) based on one-way ANOVA. Statistically significant differences based on two-way ANOVA are reported as follows: (**A**) **F%** treatment NS, time ***, treatment × time NS; (**B**) **M%** treatment **, time ***, treatment × time **; (**C**) **A%** treatment NS, time ***, treatment × time NS; (**D**) **V%** treatment NS, time *, treatment × time NS (where * *p* < 0.05; ** *p* < 0.01; *** *p* < 0.001).

The mycorrhizal colonization of the inoculated tomato plant roots after 1 and 2 months of growth is shown in Figure 6.

Although uninoculated, the plants in CFD and CRD treatments showed a mycorrhization degree (M%) of 3.77 and 7.25, respectively. The other myc treatments (MYC and PSBA + MYC) showed similar M% (6.15 and 12.19, respectively). The other considered parameters (F %, A %, and V%) showed a similar trend. The bacterial PSBA inoculum did not affect root colonization. Evaluating the two-way ANOVA, Time could be considered the factor influencing all the analyzed parameters.

## 4. Discussion

The aim of the research presented in this paper was to evaluate the application of a bacterial-based biostimulant (*P. fluorescens* and *B. amyloliquefaciens*) in combination, or not, with the commercial inoculum Micomix and to assess the impact that these administered microorganisms have on the native microbial populations in the soil and on the production of tomatoes. Fertilization reduction induces a loss in overall production (considering marketable fruits) although the difference is not significant, but it is a significant reduction if considering marketable and non-marketable fruits. However, the addition of the inocula partially restored the production rate. This finding is in line with what has been previously reported in the literature [29] and underlines how the nutrient solubilization action operated by microorganisms improves plant nutrition, and thus, its production performance, as well. BER (blossom-end rot) is a physiological disorder usually correlated with a lack of calcium in the plant that may be due to low calcium levels in the soil, or it occurs when there are wide fluctuations in soil moisture, and the plant’s ability to take up calcium from the soil is reduced. The application of biostimulants was able to partially reduce the percentage of BER compared to CRD. Among the treatments, the lower positive impact was done by the simultaneous application of the microbial consortia and the commercial inoculum Micomix.

The experiment was carried out in a field usually used for the cultivation of horticultural plants, a field that naturally had a high basal alpha diversity, as shown by the number (476) of the observed genera. The biodiversity indices (Shannon’s and Simpson’s) can also be considered very high, about 5.2 and 0.98, respectively; in fact, in natural conditions, Shannon’s diversity index (H) ranges from 1.5 to 3.5, and it rarely reaches 4.5, while Simpson’s index is a probability, with a range from 0 to 1 [35,36]. The addition of the bacterial and Micomix lead to a significant reduction of Shannon’s and Simpson’s biodiversity indices (within each considered community), a reduction that could be attributed to the selective driver effect of the species present in the inoculum. Although in literature it is not reported that the inoculation of bacterial and fungal-based biostimulants can induce a selective effect on native microbial communities in the soil, it is known that AM fungi can alter PGPB communities by nutrient competition [37]. Moreover, bacteria associated with mycorrhizal mycelium can behave as mycorrhizal helpers and interact with both the bacterial and fungal communities in rhizosphere soil. In particular, *B. amyloliquefaciens* is reported to behave as a helper of mycorrhization by Xie and coworkers [38]. AM fungi also work synergistically with both symbiotic and free-living nitrogen-fixing bacteria [37,39]. In fact, it has been reported that double inoculation of AM fungi and nitrogen-fixing bacteria induces an increased degree of mycorrhization in spinach [39]. This finding is also confirmed by the results obtained in this work in which the combined inoculation of bacteria and Micomix induces a significantly higher degree of mycorrhization than controls (having low degree of colonization) and fungal inoculum alone, at t1. This result was different (M% comparable to control) in the second sampling, corresponding to the ripening stage. The low degree of AM colonization measured in tomato roots in this experiment could depend on two factors. Firstly, relatively high levels of N and P occurring in the soil before transplanting are known to negatively affect the AM symbiosis establishment [40]. Secondly, fruit production and ripening represent a carbon sink for the plant, which results in a reduction of the available carbohydrates for the fungal partner (thus leading to reduced colonization). These findings were previously hypothesized in tomato plants by Bona and coworkers [28,29] in plants grown in open-field conditions, similar to those used for the present experiment [28,29].

Beta diversity analysis, which describes the degree of divergence between the different analyzed microbial communities, showed that the changes in the genera were significant with respect to all the considered factors (label, time and treatment). The data showed a clear effect of the plant on the composition of the microbial communities (Figure 5B), which was probably due to the release of root exudates into the soil and also the influence of the time (without considering each treatment) corresponding to the two considered phenological stages [41]. Furthermore, the different administered treatments, with reference to Figure 5C, also lead to an increased diversity among the communities associated with tomato roots. The selective effect highlighted in the analysis of alpha diversity actually leads to a diversification of the communities associated with the inclusion of each inoculum, particularly those composed of Micomix and bacteria (PSBA + MYC).

A specific signature is highlighted by considering the microbial communities associated with the presence of the plant compared to the basal condition (t0). The high soil pH values at t0 (8.29) are associated with the composition in *Actinobacteria* such as *Gaiella*, *Nocardioides*, and *Streptomyces*, genera normally found in alkaline soils [31]. The reduction in the presence of these genera in the other labels could be associated with a locally induced change in the pH of the substrate due to the addition of inocula, fertilization and the influence of plant root exudates. This hypothesis of the influence of pH shift in determining the observed variations is also concurred in the increase of some genera associated with more acidic pH, such as *Acidibacter* and *Blastocatella* [31].

Other genera that are positively stimulated by the presence of the tomato plant belong to the nitrogen-fixing bacteria (reported as legume-nodulating bacteria), such as *Allorhizobium*, *Neorhizobium*, *Pararhizobium*, *Rhizobium*, *Bradyrhizobium*, *Devosia*, *Ensifer*, and *Mesorhizobium*. This increase could be attributed to the establishment of the rhizosphere effect induced by the presence of the tomato plant root system [42].

Still associated with the presence of the plant and thus stimulated by the rhizosphere effect, is the genus *Variovorax*, well known as able to produce and degrade auxins, thus modulating plant growth. *Variovorax* strains are also able to establish later within communities, since they are more prone to use substrates derived from bacteria instead of plants [43].

The inoculation of bacterial and/or fungal-based biostimulants (*B. amyloliquefaciens* and *P. fluorescens*/Micomix) induced significant changes in native microbial populations even though their presence within the microbial communities associated with each treatment is not detectable. This is confirmed by previous studies performed on tomato plants: 43 days after the inoculation of *Pseudomonas* and *Bacillus* strains, their presence was not detected. However, plant inoculation with these strains showed significant effects on phosphorus availability and in modulating native bacterial communities across treatments [44]. Considering the signature specifically associated to the introduction of the inocula into the native microbial communities, different genera, belonging to different phyla, were stimulated by all the applied biostimulants (*B. amyloliquefaciens*, *P. fluorescens* and Micomix): *Pseudomonadota* (*Alphaproteobacteria*—*Alsobacter*, *Amylobacter*, *Skermanella*), *Cyanobacteriota* (*Leptolyngbya*), *Acidobacteriota* (*Luteitalea*), *Actinomycetota* (*Nakamurella*), *Bacteroidota* (*Rhodocytophaga*). *Luteitalea* is the only genus previously reported as stimulated by the presence of *B. amyloliquefaciens* biofertilizer. In fact, Xue and coworkers reported that *B. amyloliquefaciens* enhanced the abundance of some genera, such as *Vicinamibacter*, *Brevitalea*, *Luteitalea*, and *Desulfonatronum* [45].

The other genera that are stimulated under these conditions in the tomato rhizosphere have never been reported to be associated with the introduction of the considered microorganisms into either the tomato rhizosphere or the rhizosphere of other plants of agricultural interest. However, their stimulation turns out to be positive as they are reported to have a beneficial effect on the soil. *Alsobacter* is reported in the rhizosphere of bok choy as a phytoremediation agent of dibutyl phthalate (DBP), a compound that can be taken up by plants and thus introduced into the food chain [46]. *Amylobacter* is a potassium-solubilizing microbe [47]. *Leptolyngbya* increased the defense capacity of tomato plants against tomato bacterial cancer (*Clavibacter michiganensis* subs. *michiganensis*) [48]. *Rhodocytophaga* is a genus often associated with soils with high organic matter content and high salinity [49]. *Skermanella* can behave as a mycorrhizal helper bacterium, occurring especially where the number of AM fungal propagules is high [50].

The inoculation of the bacterial, fungal and/or their combination inocula results in an inhibition of different genera that were most represented in the controls in the presence of the plant and at baseline without the plant (t0). The genera downregulated are belonging to different phyla: *Pseudomonadota* (*Alphaproteobacteria*—*Altererythrobacter, Bradyrhizobium*, *Devosia*, *Hirschia*, *Mesorhizobium*, *Sphingopyxis*; *Betaproteobacteria*—*Cupriavidus, Ideonella*; *Gammaprotebacteria*—*Pseudoxanthomonas*, *Tahibacter*), *Bacillota* (*Fictibacillus*), *Verrucomicrobiota* (*Luteolibacter*), *Bacterioidota* (*Ohtaekwangia*, *Pseudoflavitalea*), *Actinomycetota* (*Promicromonospora*, *Streptomyces*), *Myxococcota* (*Sorangium*). Several of these genera are dependent by nitrogen availability in soil [51], but nevertheless, the authors believe that their decrease is more to blame for the selective competition effect that may have occurred at the rhizosphere level of tomato plants.

## 5. Conclusions

The results belonging to this study highlighted the well-known effect of radical apparatus and its exudates on the native microbiota communities associated with the rhizosphere. Although a high initial biodiversity, shifts in the bacterial community composition occurred and consist mainly in: (1) increase in the abundance of bacterial genera correlated to an acidification of soil, (2) enhanced density of nitrogen-fixing microbes, known for their mycorrhization helping abilities, supported also by efficient mycorrhizal colonization in plants inoculated with the mixed inoculum (PSBA + MYC).

Consequently, the changes mediated by the plant–microbe relationships, both in space and time, can have effects on microbial community structure and activity, nutrient cycling, pH, and finally, on the plant health.

Regarding the effect of bioinocula on the native rhizosphere microbiota, a significant modulation can be observed. The differences between the microbiota composition belonging to each treatment are strongly supported, as reported by the beta-diversity values, and in particular, the co-inoculation of PGPBs and the commercial inoculum Micomix performed better than the other treatments. Overall, a “silent” effect of the bionocula was reported. The bacterial species employed as biostimulants (i.e., *Bacillus amyloliquefaciens* and *Pseduomonas fluorescens*) were not found in the analyzed microbiota, as confirmed by other studies [44]. Besides, the genera that were modulated by the addition of the bioinocula belonged to taxonomical group associated with other important agricultural plants [52,53], defining some sort of core microbiome linked to the rhizosphere and directly/indirectly influenced by consortia additions.

## Figures and Tables

**Table 1:** Soil chemical composition of the soil collected at baseline (t0).

Analyzed Compound	Results	Unit of Measure	Reference Ranges	Values
**Fertility**				
pH (in Water)	8.29	-	6.50–7.50	Very high
Electrical Conductivity	105	µS cm^−1^	200–400	Low
Organic Matter	1.69	%	1.20–2	Normal
Total Nitrogen	1.038	mg kg^−1^ sms	1.000–1.500	Normal
C/N ratio	9.44	-	10.0–15.0	Low
Available Phosphorus	19.3	mg kg^−1^	20.0–40.0	Low
Active lime	1.93	%CaCO_3_	1.50–4.00	Normal
**Microelements**				
Boron	<0.50	mg kg^−1^	0.60–1.00	Low
Iron	10.3	mg kg^−1^	4.00–10.00	High
Manganese	8.02	mg kg^−1^	1.00–5.00	Very High
Copper	6.56	mg kg^−1^	0.40–1.00	Very High
Zinc	1.34	mg kg^−1^	1.00–2.00	Normal
**Exchange complex**				
Base saturation	77.5	%	50.0–80.0	Normal
Exchangeable Potassium	0.26	meq/100 g	0.50–0.80	Low
Exchangeable Calcium	16,859	meq/100 g	8000–14,000	Very High
Exchangeable Magnesium	2.39	meq/100 g	1.50–2.50	Normal
Exchangeable Sodium	<0.05	meq/100 g	0.25–0.50	Very Low
CEC	25.2	meq/100 g	10.0–20.0	Very High
**Texture**				
Silty-Loam				

**Table 2:** Marketable and unmarketable (green) fruits number and weight/plot, and % of BERfruits. Different letters indicate statistically significant differences between the treatments (*p* < 0.05).

Treatment	Marketable Fruits		Unmarketable (Green Fruits)		Total (Marketable + Green Fruits)		Unmarketable BER Fruits
	Weight (g/plot)	Number/Plot	Weight (g/plot)	Number/Plot	Weight (g/plot)	Number/Plot	% on the Total Weight
CFD	7508.20 a	127.87 a	1908.00 a	74.27 a	9416.20 a	202.13 a	23.59 b
CRD	3021.50 a	56.11 b	1770.50 a	99.35 a	4792.00 b	155.46 a	36.20 a
MYC	6438.40 a	105.85 ab	1232.40 a	65.54 a	7670.80 ab	171.39 a	31.75 ab
PSBA	5665.40 a	104.19 ab	1892.80 a	86.33 a	7558.20 ab	190.52 a	27.44 ab
PSBA + MYC	3923.50 a	84.57 ab	1497.25 a	82.80 a	5420.75 ab	167.36 a	32.88 ab

**Table 3:** Average of firmness and °Brix of 10 fruits per plot. Different letters indicate statistically significant differences between the treatments (*p* < 0.05).

Treatment	Firmness (cm/kg)	°Brix
CFD	4.11 a	5.37 a
CRD	4.16 a	5.43 a
MYC	4.46 a	5.29 a
PSBA	3.78 a	5.54 a
PSBA + MYC	4.11 a	5.49 a

**Table 4:** Data grouped by label. Percentual standardization of values vs. t0. Negative differences greater than 10% are highlighted in red, while positive differences greater than 10% are highlighted in green.

Genus	*p*-Value	FDR	t0	CFD_t1	CRD_t1	MYC_t1	PSBA_t1	PSBA + MYC_t1	CFD_t2	CRD_t2	MYC_t2	PSBA_t2	PSBA + MYC_t2	LDA Score
Acidibacter	0.00014755	0.00072408	100	155.58	159.23	154.47	227.51	147.26	199.19	261.94	174.03	155.63	145.32	3.96
Acidimicrobiia bacterium (uncultured)	0.000018	0.00017647	100	53.77	67.65	52.85	50.3	56.62	47.63	41.02	44.1	55.62	52.27	4.14
Acidobacteria bacterium (uncultured)	0.025732	0.038263	100	97.56	83.72	79.36	101.81	68.34	97.13	107.49	118.89	111.66	95.36	4.4
Acidobacteriaceae bacterium (uncultured)	0.0044007	0.0094048	100	95.95	84.66	80.08	102.73	76.22	100.34	114.27	119.35	121.2	105.64	4.53
Acidobacteriales bacterium (uncultured)	0.00047137	0.0016655	100	70	64.58	58.75	74.47	52.8	69.75	85.74	94.28	105.67	83.58	4.39
Acidovorax	0.00000469	0.0000731	100	1599.78	1408.18	3162.47	1051.46	675.82	1094.97	460.25	494.62	814.36	664.01	3.38
Acinetobacter	0.0058531	0.011751	100	3001.09	4664.02	60.65	67.34	814.12	5094.17	3764.09	6443.89	2040.25	2307.61	4.94
Actinobacterium (uncultured)	0.0013672	0.0039813	100	74	89.46	70.72	57.44	59.21	57.29	58.04	55.38	60.4	60.55	4.05
Actinomadura	0.017131	0.028373	100	81.13	82.77	85.91	84.3	89.79	67.15	70.87	59.32	52.07	70.63	2.8
Actinomycetales bacterium (uncultured)	0.00000299	0.0000529	100	97.28	114.55	97.5	76.16	96.08	70.44	57.67	61.07	79.34	98.59	3.46
Adhaeribacter	0.00047029	0.0016655	100	127.82	114.9	177.52	165.5	208.19	108.57	113.35	115.39	92.46	113.79	3.91
Aeromicrobium	0.00028085	0.0011243	100	215.74	200.93	267.55	201.62	213.57	319.23	267.17	196.75	120.57	126.37	4.61
Allorhizobium Neorhizobium Pararhizobium Rhizobium	0.0021565	0.0052715	100	167.14	182.22	177.25	145.63	188.67	247.6	221.21	209.35	194.15	176.26	4.5
Alsobacter	0.017712	0.028974	100	103.68	107.04	169.83	155.94	200.54	116.4	142.08	132.67	133.07	135.71	3.11
Altererythrobacter	0.00032195	0.0012229	100	289.68	514.48	277.11	298.3	270.04	291.05	280.58	202.53	202.07	191.35	3.65
Amaricoccus	0.035663	0.050004	100	106.47	130.89	145.28	146.72	188.74	120.64	112.51	111.01	150.5	149.04	3.2
Aminobacter	0.000000015	0.00000199	100	197.43	139.68	1110.94	1061.6	1129.53	171.17	214.17	302.93	309.59	283.42	3.53
Ammoniphilus	0.018022	0.029299	100	71.76	99.9	83.32	86.55	105.78	80.39	89.8	60.67	85.71	56.67	3.02
Amycolatopsis	0.0000000459	0.00000304	100	370.65	261.97	264.12	79.03	161.58	2499.7	752.78	1067.1	921.05	1642.91	3.71
Anaerolinea	0.024548	0.036753	100	34.12	30.49	38.59	36.77	41.71	23.45	17.9	24.69	29.63	41.04	3.61
Anaeromyxobacter	0.0023108	0.0055669	100	56.91	57.54	47.92	64.27	64.7	46.92	44.31	57.26	82.9	57.24	3.63
Arenimonas	0.0000464	0.00036396	100	264.98	231.75	240.75	216.36	225.8	281.65	204.59	113.83	120.37	113.19	3.29
Aridibacter	0.0093128	0.017138	100	172.76	134.24	167.94	159.77	192.99	146.88	144.83	121.56	113.03	159.73	3.35
Armatimonadetes bacterium (uncultured)	0.00014735	0.00072408	100	81.28	78.4	88.19	87.42	98.96	54.57	64.2	62.56	64.6	70.69	3.03
Arthrobacter	0.0066158	0.012987	100	110.14	90.11	130.95	119.67	154.37	125.5	108.41	130.33	145.57	150.85	5.28
Azohydromonas	0.000000298	0.0000132	100	156.52	148.01	179.02	154.93	222.21	323.53	325.44	271.45	244.19	192.86	3.54
Blastocatella	0.0083444	0.015683	100	165.57	142.22	175.37	141.52	165.17	133.59	124.31	131	124.01	139.93	3.5
Blastococcus	0.0061343	0.012222	100	108.48	106.89	137.17	122.74	162.49	95.23	104.31	90.25	97.41	96.46	4.28
Blastopirellula	0.0017302	0.0047761	100	96.28	91.75	84.19	112.04	79.5	127.33	149.15	178.04	181.03	143.16	3.05
Bosea	0.0011646	0.0034678	100	185.33	196.19	234.64	209.3	293.26	229.65	247.79	216.03	241.81	154.01	3.7
Bradyrhizobium	0.00045919	0.0016655	100	129.5	154.15	118.4	167.45	123.65	153.75	162.94	161.36	140.06	125.84	3.95
Burkholderia Caballeronia Paraburkholderia	0.012425	0.02138	100	46.43	60.48	54.73	46.12	43.13	71.5	65.56	32.89	29.82	28.31	3.54
Caenimonas	0.0000533	0.00038145	100	159	145.17	204.66	197.78	244.47	208.96	198.63	156.15	123.62	127.8	4.13
Candidatus Udaeobacter	0.02707	0.039854	100	95.45	90.54	58.67	65.19	41.52	82.86	95.55	95.68	89.25	92.82	4.15
Candidatus Xiphinematobacter	0.0098109	0.017686	100	91.35	73.1	72.62	87.72	64.97	97.42	116.23	128.84	122.85	120.7	4.66
Caulobacter	0.033071	0.047372	100	111.4	301.28	94.01	83.12	102.03	95.46	81.41	70.05	51.29	41.22	3.31
Cellulomonas	0.0018814	0.0050246	100	66.57	88.69	100.53	102.72	157.41	65.68	64.37	68.77	112.36	64.88	2.98
Chloroflexi bacterium (uncultured)	0.00000138	0.0000304	100	86.92	94.22	99.21	88.22	96.63	72.5	64.09	66.27	72.16	84.66	4.32
Chloroflexus sp (uncultured)	0.00000908	0.00010461	100	101.7	102.45	82.23	59.29	62.44	88.91	73.71	65.16	53.25	78.24	3.84
Chthoniobacter	0.00082646	0.0027039	100	155.64	124.66	163.29	184.98	168.04	132.46	149.85	140.19	104.27	123.15	4.28
Clostridium sensu stricto 1	0.015432	0.02572	100	55.73	63.89	48.1	56.44	62.09	44.54	30.51	60.27	50.98	48.2	3.25
Cohnella	0.0024156	0.0057671	100	64.17	79.14	73.08	82.54	97.11	42.83	52.74	50.9	82.82	74.89	3.44
Comamonas	0.00000807	0.00010186	100	199.52	199.96	203.55	221.02	189.06	422.54	364.9	324.98	335.35	229.65	3.18
Conexibacter	0.0039591	0.0088165	100	94.22	117.14	119.44	90.52	105.8	77.22	68.14	63.1	82.41	77.05	2.86
Conexibacter sp (uncultured)	0.00000683	0.0000905	100	68.43	79.04	74.4	68.73	76	47.69	41.99	46.9	58.86	63.81	2.98
Cupriavidus	0.00012448	0.0006732	100	143.59	151.65	104.49	128.17	189.52	199.9	420.69	237.72	212.86	201.48	3.53
Desulfuromonadales bacterium (uncultured)	0.033797	0.047965	100	65.76	59.63	61.14	96.03	76.67	67.29	65.58	67.82	92.78	91.59	3.17
Devosia	0.0000096	0.00010605	100	226.22	319.51	225.4	271.28	210.97	234.93	216.19	169.16	174.26	148.16	4.34
Domibacillus	0.01899	0.030499	100	90.7	78.89	69.59	81.07	108.49	160.57	129.7	152.24	156.85	235.77	3.37
Dongia	0.00020379	0.00090009	100	70.74	54.21	48.68	54.72	44.35	60.5	74.36	73.15	64.36	62.84	3.83
Dyadobacter	0.0016816	0.0046909	100	232.62	226.93	196.06	117.61	161.45	597.49	204.79	493.9	216.82	199.51	3.82
Ensifer	0.0000334	0.0002946	100	154.57	144.99	148.37	136.6	166.19	201.05	210.7	228.49	191.3	195.35	3.92
Enterobacter	0.0041214	0.0090263	100	508.34	66.44	68.61	37.4	107.23	2294.43	390.86	435.46	1041.92	391.72	4.28
Ferruginibacter	0.022231	0.033858	100	131.11	117.94	152.2	151.21	158.63	105.22	111.4	113.77	94.65	101.97	3.33
Fictibacillus	0.0000701	0.00048619	100	57	61.58	60.46	53.77	73.42	35.9	71.85	35.74	59.62	60.49	3.82
Fimbriiglobus	0.0021683	0.0052715	100	132.57	123.36	110.92	143.44	112.03	139.1	154.72	165.93	161.35	161.62	3.8
Flavisolibacter	0.0014198	0.0040548	100	151.91	131.26	182.15	163.17	179.1	132.66	149.47	119.44	97.85	111.18	3.84
Flavitalea	0.0000169	0.00017193	100	181.78	163.92	233.88	214.95	202.73	218.18	263.68	234.51	197.94	195.31	3.3
Gaiella	0.000000204	0.0000108	100	68.41	81.42	75.03	61.98	69.54	51.04	42.22	45.04	57.89	61.91	5.06
Gaiella sp (uncultured)	0.00000393	0.0000652	100	77.81	87.76	97.13	65.55	91.79	58.1	46.84	54.62	69.69	72.27	3.99
Gemmata	0.01289	0.022038	100	116.85	107.42	86.95	105.48	83.21	111.89	125.15	125.91	119.25	127.67	4.41
Gemmatimonadalesbacterium (uncultured)	0.0026426	0.0061971	100	82	70.69	51.2	67.43	41.47	65.43	75.35	76.76	83.05	81.29	3.46
Gemmatimonadetesbacterium (uncultured)	0.034256	0.048287	100	107.82	92.29	115.96	142.77	89.71	129.7	165.71	150.98	115.88	105.49	3.47
Gemmatimonas	0.00011975	0.00066221	100	167.46	134.25	176.39	187.47	155.72	175.34	190.87	179.69	140.62	139.7	3.52
Haliangium	0.019262	0.03075	100	142.07	150.98	178.47	160.7	100.56	126.82	138.32	128.47	70.12	83.26	3.66
Herbinix	0.010326	0.018366	100	59.77	69.48	54.16	58.89	75.81	44.86	44.65	36.5	68.65	55.66	2.88
Herpetosiphon	0.021416	0.032805	100	153	170.49	216.45	139.36	189.54	188.93	132.95	123.08	124.75	125.94	3.88
Hirschia	0.00000632	0.0000905	100	344.79	525.52	408.98	682.66	418.08	373.67	1232.23	424.42	210.06	180.65	3.82
Hyphomicrobium	0.01002	0.017942	100	73.17	71.68	66.73	67.52	58.92	86.64	78.82	96.11	108.3	97.81	3.04
Iamia	0.039147	0.0546	100	95.31	106.8	94.52	76.34	72.34	107.42	83.82	70.76	72.27	74.64	3.64
Iamia sp (uncultured)	0.001423	0.0040548	100	161.63	150.05	203.71	144.97	155.12	144.88	81.65	81.53	69.71	71.87	2.93
Ideonella	0.0040052	0.0088447	100	356.9	919.34	392.73	310.44	422.8	479.36	581.88	567.97	277.46	332.01	3.16
Ilumatobacter	0.047015	0.063566	100	78.54	99.56	70.17	55.72	60.49	75.65	50.63	59.65	68.94	79.38	3.47
Knoellia	0.0021669	0.0052715	100	90.33	87.36	96.59	90.73	110.48	81.06	70.72	69.84	94.59	80.69	4.4
Kribbella	0.00081255	0.0026916	100	71.11	83.71	70.86	65.83	71.24	84.09	70.24	74.46	79.79	83.96	3.05
Lacibacter	0.011803	0.020486	100	245.01	200.19	252.26	185.76	187.34	276.88	199.35	186.66	127.6	128.52	3.24
Lechevalieria	0.00000228	0.0000432	100	173.32	190.25	120.07	95.16	113.47	331.88	211.27	179.01	192.24	195.11	3.98
Leptolyngbya EcFYyyy 00	0.0000139	0.00014736	100	2312.27	5119.22	9376.78	6632.3	7496.83	1967.75	2638.27	5665.63	1643.89	1494.45	4.59
Litorilinea	0.043669	0.05996	100	121.16	126.57	136.59	151.58	166.85	121.76	110.6	119.61	105.13	134.46	3.4
Luedemannella	0.0000735	0.00048692	100	39.99	59.94	47.15	51.28	60.53	39.02	30.94	39.17	60.62	45.17	3.45
Luteitalea	0.0052232	0.01073	100	118.55	124.73	140.83	154.58	167.95	131.97	144.26	140.13	193.24	129.56	3.42
Luteolibacter	0.0000482	0.00036526	100	190.94	284.65	204.06	210.41	190.7	219.9	179.66	170.08	127.91	125.81	3.71
Lutispora sp (uncultured)	0.0043214	0.0093867	100	32.62	31.32	41.91	40.8	40.63	23.19	19.5	29.28	41.92	37.77	3.64
Lysobacter	0.0045532	0.0095762	100	135.39	128.72	160.92	118.34	150.98	146.07	132.24	102.15	106.22	100.25	3.91
Marmoricola	0.0025191	0.0059603	100	80.34	74.33	82.91	73.03	85.95	75.16	82.11	65.41	68.57	62.57	4.19
Massilia	0.031063	0.044982	100	104.7	100.21	140.59	126.46	215.03	110.64	117.27	99.62	84.87	109.16	4.54
Mesorhizobium	0.00011549	0.00066221	100	184.16	282.23	208.63	273.02	212.63	207.48	276.47	199.8	196.65	177.18	4.18
Methylobacillus	0.00000104	0.0000251	100	1748.09	1936.43	1955.12	899.75	1931.16	10944.59	3216.56	5335.2	3268.91	5021.76	3.5
Methylorosula	0.00028426	0.0011243	100	134.35	130.99	182.81	175.05	196.27	99.66	113.49	93.63	86.59	103.86	2.77
Methylotenera	0.00084022	0.0027153	100	389.84	409.42	404.84	332.11	443.43	1665.5	553.08	767.07	496.42	582.81	3.65
Microbacterium	0.001128	0.0033969	100	209.17	180.34	181.68	122.68	171.04	426.61	263.56	226.4	389.73	240.8	4.02
Microvirga	0.011174	0.019741	100	113.12	98.59	148.99	132.61	173.22	129.73	167.03	130.26	116.6	130.67	4.68
Mitsuaria	0.000000014	0.00000199	100	2471.17	4603.04	1612.26	379.1	517.39	15332.6	3375.88	18722.49	6035.04	6936.55	4.18
Mycobacterium	0.0000303	0.00028643	100	77.44	87.4	84.17	77.02	76.92	76.92	85.46	66.59	62.03	72.73	3.82
Niastella	0.0001624	0.00077527	100	235.05	288.29	234.39	236.87	235.51	370.63	314.42	385.19	312.56	284.5	3.08
Nitrospira	0.0045207	0.0095762	100	136.34	128.62	140.07	170.61	130.4	121.19	153.19	159.19	161.09	158.07	4.33
Nocardioides	0.00032053	0.0012229	100	85.8	89.32	88.58	69.58	83.16	76.51	56.98	59.01	80.5	67.38	4.35
Nodosilinea PCC 7104	0.0001395	0.00072068	100	2253.73	8243.55	3699.69	6425.11	12123.84	6449.88	4061.03	6972.14	3939.8	5529.82	3.94
Nonomuraea	0.025846	0.038263	100	60.89	82.72	78.36	73.94	91	57	47.39	64.38	77.75	68.59	3
Nordella	0.018708	0.030229	100	130.16	112.77	99.28	114.07	83	118.22	140.1	136.73	123.43	111.16	3.91
Ohtaekwangia	0.0010564	0.0032456	100	244.28	339.48	248.08	235.26	183.89	271.33	267.43	185.08	152.59	153.66	3.58
Oligoflexus	0.011473	0.020134	100	132.57	152.11	260.23	195.51	326.72	125.28	131.03	126.88	125.38	111.83	3.21
Paenarthrobacter	0.030718	0.044726	100	129.65	76.49	102.87	87.34	125.46	216.98	106.39	160.56	183.42	174.4	3.83
Paenisporosarcina	0.0077201	0.014613	100	84.37	77.24	75.17	76.1	102.17	48.46	73.1	52.54	79.84	77.1	3.51
Parasegetibacter	0.00018969	0.00085201	100	204.92	218.3	294.66	222.86	361.11	195.78	173.93	204.47	172.61	160.28	3.45
Parviterribacter	0.017589	0.028952	100	87.33	94.87	99.83	83.27	104.38	69.24	71.59	58.34	69.31	56.79	3.03
Paucibacter	0.014338	0.024202	100	284.78	208.85	265.14	247.19	257.97	217.79	224.76	185.15	171.78	179.84	2.8
Pedobacter	0.00021864	0.00093453	100	164.82	143.39	199.91	183.08	259.43	116.46	114.2	106.46	110.47	137.43	3.81
Pedococcus Phycicoccus	0.0087706	0.016368	100	85.21	89.04	86.24	61	70.52	82.17	55.39	54.03	94.25	77.84	2.75
Pedomicrobium	0.00069317	0.0023252	100	71.75	74.73	66.39	82.76	60.34	83.97	97.89	100.87	104.39	86.8	3.79
Pelobacter sp (uncultured)	0.011828	0.020486	100	122.6	102.05	122.5	107.26	59.33	125.71	192.02	199.04	110.51	90.47	2.92
Phenylobacterium	0.0045993	0.0095969	100	89.49	105.03	102.19	93.27	115.14	72.43	68.3	63.98	74.15	72.89	2.75
Pirellula	0.014538	0.024383	100	130.33	110.51	96.29	103.92	76.54	132.36	141.09	136.47	117.04	127.69	4.56
Planctomicrobium	0.0015673	0.0044184	100	188.68	156.59	131.19	142.59	87.73	264.15	215.38	197.44	150.77	200.12	2.97
Planctomycetales bacterium (uncultured)	0.00023737	0.00098821	100	192.28	167.13	173.3	210.72	122.1	285.77	298.34	247.74	193.15	258.19	2.96
Planctomycete (uncultured)	0.000033	0.0002946	100	199.24	149.86	189.05	213.02	166.87	225.66	248.26	194.23	134.27	162.06	4.33
Polaromonas	0.009301	0.017138	100	179.46	167.51	133.39	182.9	144.3	128.52	118.78	129.68	117.05	100.35	2.77
Polyangium brachysporum group	0.000000784	0.0000208	100	268.51	232.62	326.15	205.78	221.01	619.71	707.05	324.78	356.17	395.77	3.66
Pontibacter	0.0094018	0.017138	100	173.16	147.9	236.04	176.91	330.57	146.09	123.67	125.21	115.82	133.43	3.95
Promicromonospora	0.024173	0.036397	100	52.37	83.01	39.49	53.33	62.08	88.85	101.5	58.59	50.56	57.65	3.26
Pseudarthrobacter	0.0053672	0.010857	100	117.69	91.15	163.45	118.36	171.83	152.47	105.46	136.53	157.57	145.83	3.1
Pseudoduganella	0.00011	0.00066221	100	672.85	586.11	1138.12	411.03	310.16	834.46	670.35	770.86	412.84	450.85	3.55
Pseudoflavitalea	0.0021239	0.0052715	100	156.52	170.9	125.18	286.59	115.08	92.09	290.91	162.12	105.56	107.96	2.97
Pseudolabrys	0.0050874	0.010532	100	121.48	186.31	90.15	145.14	85.18	85.56	91.97	75.5	73.53	76.44	3.87
Pseudomuriella schumacherensis	0.00011443	0.00066221	100	1106.64	1024.92	1188.77	1364.16	1518.29	548.54	893.81	1066.76	659.19	692.24	3.48
Pseudorhodoplanes	0.00213	0.0052715	100	179.58	201.33	186.53	260.72	168.81	158.37	237.17	206.26	181.6	167.52	3.02
Pseudoxanthomonas	0.00011106	0.00066221	100	253.63	454.14	234.01	173.62	201.24	559.88	403.38	381.86	291.59	216.07	3.99
Qipengyuania	0.00000207	0.0000422	100	297.75	257.97	339.14	191.27	277.65	180.67	116.69	119.76	138.04	134.82	3.83
Ramlibacter	0.00000652	0.0000905	100	181.28	161	236.7	219.99	277.08	265.84	273.63	218.76	141.41	154.73	4.08
Reyranella	0.004395	0.0094048	100	126.84	137.28	113.72	150.05	97.42	137.09	163.7	166.75	117.23	122.19	3.35
Rhizobacter	0.0000521	0.00038145	100	174.57	197.02	194.21	191.55	218.94	223.06	200.44	179.82	164.11	150.41	3.96
Rhodobacter	0.0000467	0.00036396	100	221.61	342.49	418.73	396.49	466.8	204.58	122.81	136.38	134.34	109.9	3.52
Rhodocytophaga	0.028682	0.041993	100	191.82	167.95	210.05	187.24	253.2	196.79	192.47	238.35	217.3	225.2	3.21
Rhodopirellula	0.0000374	0.00031979	100	286.18	240.7	315.43	257.59	351.41	280.86	318.8	218.5	178.75	202.5	3.71
Rhodoplanes	0.040253	0.055849	100	124.38	115.81	90.29	108.79	73.07	125.18	129.23	146.31	125.73	116.69	3.69
Roseimicrobium	0.046453	0.063129	100	127.53	103.51	116.26	131.14	79.08	131.94	123.46	126.8	95.22	102.17	3.29
Roseomonas	0.0028822	0.0065843	100	152.77	155.58	216.23	213.38	317.78	160.55	177.73	154.02	144.78	130.02	3.18
Rubellimicrobium	0.00097379	0.0030721	100	180.34	153.55	261.22	232.54	350.73	187.62	165.95	162.6	165.33	146.86	4.16
Rubrobacter	0.0094423	0.017138	100	92.81	99.13	115.01	104.29	122.75	77.76	85	83.38	83.85	96.1	4.52
Rubrobacterales bacterium (uncultured)	0.0000000363	0.00000304	100	70.11	79.72	65.57	53.54	55.68	47.42	39.19	38.75	48.12	60.7	4.44
Rubrobacteria bacterium (uncultured)	0.000000765	0.0000208	100	75.13	92.58	78.42	61.37	73.87	54.54	44.77	48.19	60.06	68.59	3.93
Saccharothrix	0.031572	0.04547	100	176.51	131.74	138.84	138.58	199.97	120.79	80.1	84.76	73.18	98.96	3.17
Shimazuella	0.020834	0.032287	100	56.67	73.25	82.5	74.12	100.33	70.95	52.39	79.53	87.62	68.01	2.79
Shinella	0.00053039	0.0018494	100	573.73	766.54	460.95	311.9	450.67	722.3	409.51	561.55	450.4	332.76	3.6
Skermanella	0.033847	0.047965	100	93.88	103.19	127.89	151.19	176.97	98.94	97.63	100.79	119.95	111.45	4.41
Solirubrobacter	0.0000462	0.00036396	100	74.19	84.79	80.89	73.16	78.94	57	53.45	53.21	70.25	63.61	3.97
Sorangium	0.0021467	0.0052715	100	46.76	115.95	56.46	55.15	41.56	128.87	164.06	69.81	79.75	51.39	3.08
Sphingoaurantiacus	0.00023866	0.00098821	100	137.24	127.25	188.94	156.67	253.86	113.44	105.64	80.63	104.08	109.26	3.22
Sphingobium	0.00000846	0.00010189	100	1535.96	1807.61	2282.28	1206.9	1949.55	4110.56	2082.64	2135.58	1950.4	1651.48	4.7
Sphingomonas	0.00025341	0.0010331	100	113.23	115.85	128.93	95.94	124.51	98.88	96.23	77.57	84.93	94.5	4.65
Sphingopyxis	0.00011995	0.00066221	100	336.67	528.52	344.6	267.99	391.86	330.81	114.14	152.5	170.98	211.84	3.34
Steroidobacter	0.00032304	0.0012229	100	77.88	89.19	80.14	103.14	84.97	104.13	123.26	123.89	137.99	106.49	3.9
Streptomyces	0.00053812	0.001852	100	143.71	147.41	108.38	114.58	117.28	169.34	147.64	120.62	137.28	137.42	4.2
Sumerlaea	0.0010606	0.0032456	100	202.52	154.59	213.89	210.88	208.49	155.3	203.34	189.77	170.64	133.07	3.09
Synechococcus IR11	0.0018714	0.0050246	100	2059.31	8772.95	7010.01	9186.14	13235.59	3336.4	9873.98	3522.57	3217.56	4680.08	3.55
Tahibacter	0.0067223	0.013003	100	325.35	250.32	201	152.21	171.68	456.93	513.45	240.03	191.08	242.91	3.09
Tepidisphaera	0.00308	0.0069668	100	192.6	131.89	222.59	225.5	186.88	151.91	164.68	156.13	83.33	98.18	3.19
Terrimonas	0.0031022	0.0069668	100	173.55	162.97	160.76	177.85	132.57	193.79	238.46	200.37	140.34	137.5	3.85
Thermomicrobia bacterium (uncultured)	0.041362	0.057088	100	92.53	105.02	86.79	97.95	114.08	91.75	71.3	72.09	74.38	96.51	2.63
Truepera	0.000000551	0.0000198	100	246.02	169.5	255.84	302.82	315.02	698.33	970.88	831.76	967.32	617.8	3.55
Tumebacillus	0.0018961	0.0050246	100	48.55	94.68	54.44	65.91	86.44	36.34	28.91	46.08	103.41	69.26	3.71
Tychonema CCAP 1459 11B	0.0066796	0.013003	100	369.76	335.37	240.12	439.63	398.29	132.46	88.47	234	161.77	141.96	4.09
Variovorax	0.0000987	0.0006228	100	164.4	186.5	126.31	131.17	138.76	283.49	193.64	207.47	174.6	124.95	3.81
Yonghaparkia	0.000000596	0.0000198	100	879.56	846.51	1136.14	574.27	1120.71	294.83	186.43	200.65	247.47	271.22	3.71

**Table 5:** Data grouped by time. Percentual standardization of values vs. t0. Negative differences greater than 10% are highlighted in red, while positive differences greater than 10% are highlighted in green.

Genus	*p*-Value	FDR	t0	t1	t2	LDA Score
Acidibacter	0.00016	0.00041	100	168.63	188.29	3.69
Acidimicrobiia bacterium (uncultured)	0.00000	0.00000	100	56.33	**48.02**	4.09
Acidobacteria bacterium (uncultured)	0.00959	0.01405	100	85.88	106.38	4.01
Acidobacteriaceae bacterium (uncultured)	0.00021	0.00051	100	87.83	112.32	4.27
Acidobacteriales bacterium (uncultured)	0.00002	0.00008	100	63.97	87.92	4.22
Acidobacterium sp (uncultured)	0.00948	0.01399	100	97.12	73.9	3.16
Acidovorax	0.00000	0.00000	100	**1517.39**	**706.69**	3.04
Acinetobacter	0.00044	0.00094	100	**1740.27**	**3971.71**	4.73
Actinobacterium (uncultured)	0.00011	0.00030	100	69.88	58.28	4.03
Actinomadura	0.00018	0.00043	100	84.88	63.84	2.67
Actinomycetales bacterium (uncultured)	0.00000	0.00002	100	96.28	72.78	3.14
Adhaeribacter	0.00009	0.00025	100	159.55	108.58	3.63
Aeromicrobium	0.00034	0.00075	100	218.53	208.06	4.34
Allorhizobium Neorhizobium Pararhizobium Rhizobium	0.00001	0.00005	100	172.47	210.57	4.37
Altererythrobacter	0.00000	0.00001	100	329.74	234.6	3.39
Aminobacter	0.00002	0.00008	100	**728.31**	255.57	3.32
Ammoniphilus	0.00586	0.00930	100	90.02	75.11	2.73
Amycolatopsis	0.00000	0.00000	100	224.91	**1369.89**	3.43
Anaerolinea	0.00078	0.00153	100	**36.41**	**26.99**	3.56
Anaeromyxobacter	0.00042	0.00091	100	58.69	57.74	3.51
Arenimonas	0.00001	0.00003	100	235.56	168.1	3.16
Aridibacter	0.00047	0.00099	100	166.17	136.63	3.2
Armatimonadetes bacterium (uncultured)	0.00000	0.00000	100	87.12	63.13	2.93
Aurantisolimonas	0.00384	0.00640	100	159.08	183.63	2.65
Azohydromonas	0.00000	0.00000	100	173.22	273.51	3.43
Blastocatella	0.00007	0.00020	100	157.71	130.33	3.38
Blastococcus	0.00027	0.00063	100	128.19	96.74	3.92
Blastopirellula	0.00001	0.00004	100	92.63	156.06	2.85
Bosea	0.00005	0.00015	100	225.21	219.49	3.52
Bradyrhizobium	0.00052	0.00109	100	138.76	149.38	3.81
Burkholderia Caballeronia Paraburkholderia	0.00151	0.00277	100	**49.89**	**46.06**	3.41
Caenimonas	0.00007	0.00022	100	191.21	163.94	3.93
Candidatus Alysiosphaera	0.04607	0.05786	100	118.65	99.14	3.23
Candidatus Solibacter	0.00510	0.00824	100	70.93	82.45	3.01
Candidatus Udaeobacter	0.01891	0.02544	100	69.85	91.19	3.86
Candidatus Xiphinematobacter	0.00013	0.00033	100	77.76	117.12	4.45
Caulobacter	0.00712	0.01103	100	138.57	68.57	2.74
Cellulomonas	0.01327	0.01870	100	104.61	75.48	2.47
Chloroflexi bacterium (uncultured)	0.00000	0.00000	100	92.98	71.61	4.22
Chloroflexus sp (uncultured)	0.00419	0.00695	100	81.13	71.69	3.6
Chthoniobacter	0.00013	0.00033	100	159.44	130.16	4.13
Chthonomonas	0.00694	0.01089	100	140.14	124.96	2.43
Clostridium sensu stricto 1	0.00065	0.00132	100	57.6	**46.87**	3.13
Clostridium sensu stricto 13	0.00240	0.00424	100	77.34	58.68	2.64
Clostridium sensu stricto 8	0.00433	0.00712	100	66.59	54.98	2.77
Cohnella	0.00089	0.00168	100	79.81	60.48	3.28
Comamonas	0.00000	0.00000	100	202.26	338.2	3.05
Conexibacter	0.00004	0.00012	100	105.08	73.49	2.61
Conexibacter sp (uncultured)	0.00000	0.00000	100	73.36	51.54	2.9
Cupriavidus	0.00001	0.00003	100	145.61	255.89	3.21
Cyanobacterium sp (uncultured)	0.01007	0.01467	100	157.02	79.88	3.01
Desmochloris halophila	0.00000	0.00000	100	**3208.47**	**1996.35**	3.27
Desulfuromonadales bacterium (uncultured)	0.03356	0.04317	100	72.23	76.64	3.01
Devosia	0.00000	0.00000	100	250.31	189.57	4.18
Domibacillus	0.00023	0.00055	100	86.72	165.26	3.05
Dongia	0.00001	0.00003	100	54.43	67.15	3.74
Dyadobacter	0.00061	0.00124	100	186.07	346.17	3.52
Ensifer	0.00000	0.00000	100	150.59	205.63	3.84
Enterobacter	0.00005	0.00015	100	158.57	**924.19**	3.84
Ferrimicrobium sp (uncultured)	0.01624	0.02254	100	74.34	83.52	2.8
Ferruginibacter	0.00054	0.00112	100	142.38	105.49	3.15
Fictibacillus	0.00002	0.00006	100	61.57	52.52	3.69
Fimbriiglobus	0.00001	0.00006	100	124.49	156.41	3.73
Flavisolibacter	0.00033	0.00074	100	161.44	122.4	3.71
Flavitalea	0.00000	0.00001	100	198.67	222.61	3.17
Gaiella	0.00000	0.00000	100	71.14	51.35	4.99
Gaiella sp (uncultured)	0.00000	0.00000	100	83.87	60	3.86
Galbitalea	0.03930	0.04983	100	97.77	71.06	3
Gemmata	0.00113	0.00213	100	99.89	121.83	4.1
Gemmatimonadales bacterium (uncultured)	0.00020	0.00049	100	62.32	76.25	3.27
Gemmatimonadetes bacterium (uncultured)	0.02065	0.02763	100	109.05	134.27	3.12
Gemmatimonas	0.00003	0.00009	100	163.74	165.9	3.39
Geodermatophilus	0.02480	0.03286	100	141.78	120.65	2.4
Haliangium	0.03644	0.04665	100	144.6	110.07	3.28
Herbinix	0.00058	0.00119	100	64.16	**49.92**	2.78
Herpetosiphon	0.00144	0.00265	100	173.1	139.47	3.68
Hirschia	0.00000	0.00000	100	476.23	491.99	3.36
Hyphomicrobium	0.00011	0.00030	100	67.41	93.43	2.86
Iamia sp (uncultured)	0.00004	0.00013	100	161.88	90.39	2.66
Ideonella	0.00006	0.00017	100	481.19	450.69	2.83
Ilumatobacter	0.00477	0.00775	100	72.65	66.53	3.3
Intrasporangium	0.01812	0.02475	100	89.74	71.47	2.72
Knoellia	0.00037	0.00081	100	95.44	79.35	4.1
Kribbella	0.00008	0.00024	100	72.56	78.37	2.96
Lacibacter	0.00466	0.00763	100	212.49	185.22	3.05
Lechevalieria	0.00000	0.00000	100	138.29	222.59	3.7
Leptolyngbya EcFYyyy 00	0.00000	0.00000	100	**6140.49**	**2712.46**	4.4
Litorilinea	0.00832	0.01254	100	141.3	117.9	3.19
Luedemannella	0.00001	0.00004	100	52.11	**42.93**	3.37
Luteitalea	0.00951	0.01399	100	142.01	148.3	3.14
Luteolibacter	0.00000	0.00001	100	215.82	165.67	3.51
Lutispora sp (uncultured)	0.00033	0.00074	100	**37.42**	**30.14**	3.58
Lysobacter	0.00127	0.00235	100	138.62	117.82	3.71
Marmoricola	0.00004	0.00012	100	79.39	70.97	4.08
Massilia	0.03110	0.04021	100	139.26	104.19	4.02
Mesorhizobium	0.00000	0.00001	100	232.23	212.4	4.04
Methylobacillus	0.00000	0.00000	100	**1693.52**	**5571.14**	3.21
Methylorosula	0.00000	0.00003	100	164.23	99.33	2.54
Methylotenera	0.00001	0.00004	100	396.89	**818.87**	3.31
Microbacterium	0.00001	0.00004	100	172.71	311.19	3.83
Microvirga	0.03040	0.03969	100	133.91	134.97	4.35
Mitsuaria	0.00000	0.00000	100	**1889.22**	**10161.07**	3.91
Modestobacter	0.01267	0.01804	100	131.43	105.42	2.74
Mycobacterium	0.00001	0.00005	100	80.41	72.75	3.67
Niastella	0.00000	0.00000	100	246.05	334.72	2.99
Nitrosospira	0.01857	0.02523	100	214	140.23	2.88
Nitrospira	0.00069	0.00139	100	140.96	150.35	4.18
Nocardia	0.00690	0.01089	100	164.31	195.6	2.73
Nocardioides	0.00001	0.00004	100	83.15	68.1	4.22
Nodosilinea PCC 7104	0.00000	0.00001	100	**6759.82**	**5387**	3.68
Nonomuraea	0.00570	0.00910	100	77.7	62.88	2.84
Nordella	0.01450	0.02033	100	107.45	126.31	3.58
Ohtaekwangia	0.00011	0.00030	100	248.6	207.36	3.37
Oligoflexus	0.00231	0.00412	100	215.09	124.39	2.92
Paenarthrobacter	0.02146	0.02858	100	104.93	168.2	3.52
Paenisporosarcina	0.00367	0.00615	100	83.68	65.93	3.32
Parasegetibacter	0.00000	0.00001	100	262.03	181.96	3.24
Parviterribacter	0.00009	0.00025	100	94.05	65.27	2.89
Paucibacter	0.00028	0.00065	100	252.6	196.28	2.71
Pedobacter	0.00000	0.00001	100	191.62	116.48	3.57
Pedococcus Phycicoccus	0.00348	0.00594	100	78.01	72.61	2.53
Pedomicrobium	0.00002	0.00006	100	71.04	94.99	3.6
Pelobacter sp (uncultured)	0.01273	0.01804	100	101.17	144.91	2.43
Phenylobacterium	0.00001	0.00005	100	101.35	70.29	2.53
Phyllobacterium	0.00854	0.01279	100	142.54	161.39	3.23
Pirellula	0.00358	0.00609	100	103.02	131.02	4.24
Planctomicrobium	0.00024	0.00057	100	140.27	205.71	2.74
Planctomycetales bacterium (uncultured)	0.00000	0.00003	100	171.82	256.6	2.86
planctomycete (uncultured)	0.00009	0.00024	100	183.06	193.69	4.13
Polaromonas	0.00012	0.00031	100	161.78	119.35	2.64
Polyangium brachysporum group	0.00000	0.00000	100	248.18	482.87	3.46
Polycyclovorans	0.00710	0.01103	100	118.08	62.03	2.94
Pontibacter	0.00013	0.00034	100	215.28	128.73	3.64
Promicromonospora	0.01552	0.02164	100	58.62	71.79	3.09
Pseudoduganella	0.00002	0.00007	100	**602.96**	**632.41**	3.26
Pseudolabrys	0.00329	0.00570	100	125.53	80.71	3.47
Pseudomuriella schumacherensis	0.00000	0.00000	100	**1248.8**	**774.15**	3.38
Pseudonocardia	0.00768	0.01183	100	89.75	73.45	2.79
Pseudorhodoplanes	0.00008	0.00022	100	198.95	190.77	2.81
Pseudoxanthomonas	0.00002	0.00008	100	262.52	374.52	3.77
Qipengyuania	0.00000	0.00000	100	271.22	138.08	3.68
Ramlibacter	0.00001	0.00004	100	216.22	212.31	3.9
Reyranella	0.01073	0.01554	100	124.65	141.88	3.13
Rhizobacter	0.00000	0.00003	100	195.87	184.42	3.85
Rhodobacter	0.00000	0.00000	100	370.43	142.41	3.39
Rhodocytophaga	0.00081	0.00156	100	203.13	213.74	3.08
Rhodopirellula	0.00000	0.00001	100	291.17	240.84	3.59
Rhodoplanes	0.01165	0.01668	100	102.04	128.93	3.28
Risungbinella	0.04659	0.05824	100	87.62	88.28	2.1
Roseomonas	0.00025	0.00058	100	213.69	154.02	2.9
Rubellimicrobium	0.00005	0.00015	100	237.91	166.15	3.9
Rubrobacter	0.00160	0.00289	100	106.99	84.94	4.22
Rubrobacterales bacterium (uncultured)	0.00000	0.00000	100	64.68	**46.48**	4.38
Rubrobacteria bacterium (uncultured)	0.00000	0.00000	100	76.16	54.89	3.84
Saccharothrix	0.00049	0.00104	100	158.65	91.37	2.89
Shinella	0.00001	0.00004	100	**512.5**	499.48	3.39
Solirubrobacter	0.00000	0.00000	100	78.34	59.4	3.91
Sorangium	0.00117	0.00219	100	62.8	99.99	2.57
Sphingoaurantiacus	0.00000	0.00003	100	174.41	102.44	2.85
Sphingobium	0.00000	0.00001	100	**1748.14**	**2404.97**	4.46
Sphingomonas	0.00002	0.00008	100	115.58	90.32	4.34
Sphingopyxis	0.00002	0.00008	100	375.11	195.65	3.14
Sporacetigenium	0.04686	0.05829	100	89.43	76.16	2.54
Stenotrophobacter	0.00870	0.01296	100	123.55	97.76	2.6
Steroidobacter	0.00001	0.00005	100	87.18	119.48	3.63
Streptomyces	0.00085	0.00162	100	126.49	142.59	3.99
Subgroup 10	0.00005	0.00015	100	184.27	160.54	3.92
Sumerlaea	0.00006	0.00017	100	197.94	171.38	3.02
Synechococcus IR11	0.00003	0.00011	100	**8208.54**	**4932.48**	3.34
Syntrophobacterales bacterium (uncultured)	0.03018	0.03959	100	104.28	130.87	2.24
Tahibacter	0.00155	0.00282	100	219.39	331.07	2.84
Tepidisphaera	0.00362	0.00611	100	191	131.68	3
Terrimonas	0.00084	0.00162	100	160.84	183.24	3.63
Thermomicrobia bacterium (uncultured)	0.00774	0.01185	100	99.96	80.82	2.29
Truepera	0.00000	0.00000	100	259.32	**822.33**	3.47
Tumebacillus	0.01690	0.02332	100	70.8	56.48	3.48
Tychonema CCAP 1459 11B	0.00003	0.00011	100	360.59	151.98	3.96
Variovorax	0.00004	0.00012	100	149.74	198.67	3.54
Yonghaparkia	0.00000	0.00000	100	**911.04**	239.32	3.6

**Table 6:** Data grouped by label. Percentual standardization of values vs. CFD_t1 or CFD_t2. Negative differences greater than 10% are highlighted in red, while positive differences greater than 10% are highlighted in green.

Genus	*p*-Value	FDR	t0	CFD_t1	CRD_t1	MYC_t1	PSBA_t1	PSBA + MYC_t1	CFD_t2	CRD_t2	MYC_t2	PSBA_t2	PSBA + MYC_t2	LDA Score
Acidibacter	0.00015	0.00072	64.28	100	102.35	99.28	146.23	94.65	100	131.51	87.37	78.13	72.96	3.96
Acidimicrobiia bacterium (uncultured)	0.00002	0.00018	185.98	100	125.83	98.29	93.56	105.3	100	86.13	92.59	116.77	109.73	4.14
Acidobacteria bacterium (uncultured)	0.02573	0.03826	102.5	100	85.82	81.35	104.36	70.05	100	110.67	122.39	114.96	98.17	4.4
Acidobacteriaceae bacterium (uncultured)	0.00440	0.00940	104.22	100	88.24	83.46	107.07	79.43	100	113.88	118.94	120.78	105.28	4.53
Acidobacteriales bacterium (uncultured)	0.00047	0.00167	142.86	100	92.26	83.92	106.38	75.43	100	122.93	135.17	151.49	119.83	4.39
Acidovorax	0.00000	0.00007	6.25	100	88.02	197.68	65.73	42.24	100	42.03	45.17	74.37	60.64	3.38
Acinetobacter	0.00585	0.01175	3.33	100	155.41	2.02	2.24	27.13	100	73.89	126.5	40.05	45.3	4.94
Actinobacterium (uncultured)	0.00137	0.00398	135.13	100	120.88	95.57	77.62	80.01	100	101.31	96.68	105.43	105.69	4.05
Actinomadura	0.01713	0.02837	123.26	100	102.02	105.9	103.91	110.68	100	105.54	88.34	77.54	105.18	2.8
Actinomycetales bacterium (uncultured)	0.00000	0.00005	102.79	100	117.75	100.22	78.29	98.77	100	81.87	86.69	112.63	139.96	3.46
Adhaeribacter	0.00047	0.00167	78.24	100	89.89	138.89	129.48	162.88	100	104.4	106.29	85.17	104.81	3.91
Aeromicrobium	0.00028	0.00112	46.35	100	93.14	124.02	93.46	99	100	83.69	61.63	37.77	39.58	4.61
Allorhizobium Neorhizobium Pararhizobium Rhizobium	0.00216	0.00527	59.83	100	109.03	106.05	87.13	112.88	100	89.34	84.55	78.41	71.19	4.5
Alsobacter	0.01771	0.02897	96.45	100	103.24	163.8	150.41	193.42	100	122.06	113.97	114.31	116.59	3.11
Altererythrobacter	0.00032	0.00122	34.52	100	177.6	95.66	102.98	93.22	100	96.4	69.59	69.43	65.75	3.65
Amaricoccus	0.03566	0.05000	93.92	100	122.93	136.45	137.8	177.26	100	93.26	92.02	124.75	123.54	3.2
Aminobacter	0.00000	0.00000	50.65	100	70.75	562.69	537.69	572.1	100	125.13	176.98	180.87	165.58	3.53
Ammoniphilus	0.01802	0.02930	139.35	100	139.21	116.1	120.61	147.41	100	111.7	75.46	106.62	70.49	3.02
Amycolatopsis	0.00000	0.00000	26.98	100	70.68	71.26	21.32	43.59	100	30.11	42.69	36.85	65.72	3.71
Anaerolinea	0.02455	0.03675	293.06	100	89.34	113.08	107.76	122.25	100	76.34	105.3	126.33	175	3.61
Anaeromyxobacter	0.00231	0.00557	175.72	100	101.11	84.21	112.93	113.7	100	94.44	122.04	176.69	121.99	3.63
Arenimonas	0.00005	0.00036	37.74	100	87.46	90.86	81.65	85.21	100	72.64	40.42	42.74	40.19	3.29
Aridibacter	0.00931	0.01714	57.89	100	77.7	97.22	92.48	111.72	100	98.61	82.76	76.95	108.75	3.35
Armatimonadetes bacterium (uncultured)	0.00015	0.00072	123.04	100	96.46	108.5	107.56	121.76	100	117.64	114.64	118.38	129.54	3.03
Arthrobacter	0.00662	0.01299	90.79	100	81.82	118.9	108.65	140.16	100	86.38	103.85	115.99	120.2	5.28
Azohydromonas	0.00000	0.00001	63.89	100	94.56	114.38	98.98	141.97	100	100.59	83.9	75.48	59.61	3.54
Blastocatella	0.00834	0.01568	60.4	100	85.9	105.92	85.48	99.76	100	93.05	98.06	92.82	104.74	3.5
Blastococcus	0.00613	0.01222	92.19	100	98.54	126.45	113.14	149.79	100	109.53	94.77	102.3	101.3	4.28
Blastopirellula	0.00173	0.00478	103.86	100	95.29	87.44	116.36	82.57	100	117.14	139.83	142.18	112.43	3.05
Bosea	0.00116	0.00347	53.96	100	105.86	126.6	112.93	158.23	100	107.9	94.07	105.3	67.06	3.7
Bradyrhizobium	0.00046	0.00167	77.22	100	119.03	91.43	129.3	95.48	100	105.98	104.95	91.1	81.84	3.95
Burkholderia Caballeronia Paraburkholderia	0.01243	0.02138	215.37	100	130.25	117.86	99.32	92.88	100	91.69	46.01	41.7	39.6	3.54
Caenimonas	0.00005	0.00038	62.89	100	91.3	128.72	124.39	153.75	100	95.05	74.73	59.16	61.16	4.13
Candidatus Udaeobacter	0.02707	0.03985	104.76	100	94.85	61.47	68.29	43.49	100	115.31	115.47	107.71	112.02	4.15
Candidatus Xiphinematobacter	0.00981	0.01769	109.47	100	80.02	79.5	96.03	71.13	100	119.3	132.25	126.1	123.89	4.66
Caulobacter	0.03307	0.04737	89.77	100	270.46	84.39	74.62	91.59	100	85.27	73.37	53.73	43.18	3.31
Cellulomonas	0.00188	0.00502	150.21	100	133.23	151.02	154.3	236.45	100	98	104.7	171.07	98.77	2.98
Chloroflexi bacterium (uncultured)	0.00000	0.00003	115.05	100	108.39	114.14	101.5	111.16	100	88.41	91.41	99.54	116.77	4.32
Chloroflexus sp (uncultured)	0.00001	0.00010	98.32	100	100.73	80.85	58.3	61.4	100	82.9	73.29	59.88	87.99	3.84
Chthoniobacter	0.00083	0.00270	64.25	100	80.09	104.91	118.85	107.96	100	113.12	105.83	78.72	92.97	4.28
Clostridium sensu stricto 1	0.01543	0.02572	179.45	100	114.66	86.31	101.28	111.43	100	68.49	135.32	114.45	108.22	3.25
Cohnella	0.00242	0.00577	155.83	100	123.32	113.88	128.62	151.32	100	123.15	118.85	193.39	174.87	3.44
Comamonas	0.00001	0.00010	50.12	100	100.22	102.02	110.78	94.76	100	86.36	76.91	79.37	54.35	3.18
Conexibacter	0.00396	0.00882	106.13	100	124.32	126.76	96.07	112.28	100	88.24	81.71	106.72	99.78	2.86
Conexibacter sp (uncultured)	0.00001	0.00009	146.13	100	115.5	108.72	100.43	111.05	100	88.04	98.33	123.42	133.81	2.98
Cupriavidus	0.00012	0.00067	69.64	100	105.61	72.77	89.26	131.98	100	210.44	118.92	106.48	100.79	3.53
Desulfuromonadales bacterium (uncultured)	0.03380	0.04797	152.06	100	90.67	92.97	146.01	116.58	100	97.45	100.78	137.87	136.11	3.17
Devosia	0.00001	0.00011	44.2	100	141.24	99.64	119.92	93.26	100	92.02	72	74.17	63.06	4.34
Domibacillus	0.01899	0.03050	110.25	100	86.97	76.72	89.38	119.61	100	80.78	94.81	97.69	146.83	3.37
Dongia	0.00020	0.00090	141.37	100	76.64	68.82	77.36	62.7	100	122.9	120.9	106.37	103.87	3.83
Dyadobacter	0.00168	0.00469	42.99	100	97.55	84.28	50.56	69.41	100	34.27	82.66	36.29	33.39	3.82
Ensifer	0.00003	0.00029	64.7	100	93.8	95.99	88.38	107.52	100	104.8	113.65	95.15	97.16	3.92
Enterobacter	0.00412	0.00903	19.67	100	13.07	13.5	7.36	21.09	100	17.04	18.98	45.41	17.07	4.28
Ferruginibacter	0.02223	0.03386	76.27	100	89.95	116.09	115.33	120.99	100	105.87	108.12	89.95	96.91	3.33
Fictibacillus	0.00007	0.00049	175.43	100	108.02	106.06	94.32	128.8	100	200.17	99.55	166.08	168.51	3.82
Fimbriiglobus	0.00217	0.00527	75.43	100	93.06	83.67	108.2	84.51	100	111.23	119.29	115.99	116.19	3.8
Flavisolibacter	0.00142	0.00405	65.83	100	86.41	119.91	107.41	117.9	100	112.68	90.04	73.77	83.81	3.84
Flavitalea	0.00002	0.00017	55.01	100	90.17	128.66	118.24	111.52	100	120.85	107.48	90.72	89.52	3.3
Gaiella	0.00000	0.00001	146.19	100	119.03	109.68	90.61	101.66	100	82.71	88.23	113.41	121.29	5.06
Gaiella sp (uncultured)	0.00000	0.00007	128.52	100	112.79	124.84	84.24	117.97	100	80.62	94.01	119.96	124.39	3.99
Gemmata	0.01289	0.02204	85.58	100	91.93	74.41	90.27	71.21	100	111.85	112.53	106.58	114.1	4.41
Gemmatimonadalesbacterium (uncultured)	0.00264	0.00620	121.95	100	86.21	62.44	82.23	50.58	100	115.15	117.31	126.92	124.24	3.46
Gemmatimonadetesbacterium (uncultured)	0.03426	0.04829	92.74	100	85.6	107.54	132.41	83.2	100	127.76	116.41	89.34	81.33	3.47
Gemmatimonas	0.00012	0.00066	59.72	100	80.17	105.33	111.95	92.99	100	108.86	102.49	80.2	79.67	3.52
Haliangium	0.01926	0.03075	70.39	100	106.27	125.61	113.11	70.78	100	109.07	101.3	55.29	65.65	3.66
Herbinix	0.01033	0.01837	167.31	100	116.25	90.61	98.53	126.84	100	99.52	81.36	153.03	124.08	2.88
Herpetosiphon	0.02142	0.03281	65.36	100	111.43	141.47	91.08	123.88	100	70.37	65.14	66.03	66.66	3.88
Hirschia	0.00001	0.00009	29	100	152.42	118.62	197.99	121.26	100	329.77	113.58	56.22	48.35	3.82
Hyphomicrobium	0.01002	0.01794	136.67	100	97.96	91.2	92.28	80.52	100	90.97	110.93	125	112.89	3.04
Iamia	0.03915	0.05460	104.92	100	112.06	99.18	80.1	75.9	100	78.03	65.87	67.28	69.49	3.64
Iamia sp (uncultured)	0.00142	0.00405	61.87	100	92.84	126.04	89.69	95.97	100	56.36	56.27	48.11	49.61	2.93
Ideonella	0.00401	0.00884	28.02	100	257.59	110.04	86.98	118.47	100	121.39	118.48	57.88	69.26	3.16
Ilumatobacter	0.04702	0.06357	127.33	100	126.77	89.35	70.95	77.02	100	66.93	78.85	91.13	104.93	3.47
Knoellia	0.00217	0.00527	110.71	100	96.72	106.93	100.44	122.31	100	87.25	86.16	116.69	99.55	4.4
Kribbella	0.00081	0.00269	140.63	100	117.72	99.66	92.58	100.19	100	83.53	88.55	94.88	99.85	3.05
Lacibacter	0.01180	0.02049	40.81	100	81.71	102.96	75.82	76.46	100	72	67.42	46.08	46.42	3.24
Lechevalieria	0.00000	0.00004	57.7	100	109.76	69.28	54.9	65.47	100	63.66	53.94	57.93	58.79	3.98
Leptolyngbya EcFYyyy 00	0.00001	0.00015	4.32	100	221.39	405.52	286.83	324.22	100	134.08	287.92	83.54	75.95	4.59
Litorilinea	0.04367	0.05996	82.54	100	104.47	112.74	125.11	137.71	100	90.84	98.23	86.34	110.44	3.4
Luedemannella	0.00007	0.00049	250.08	100	149.9	117.91	128.24	151.38	100	79.31	100.38	155.36	115.77	3.45
Luteitalea	0.00522	0.01073	84.35	100	105.21	118.79	130.39	141.67	100	109.31	106.19	146.43	98.18	3.42
Luteolibacter	0.00005	0.00037	52.37	100	149.08	106.88	110.2	99.88	100	81.7	77.34	58.17	57.21	3.71
Lutispora sp (uncultured)	0.00432	0.00939	306.56	100	96.01	128.46	125.08	124.57	100	84.12	126.3	180.79	162.91	3.64
Lysobacter	0.00455	0.00958	73.86	100	95.08	118.86	87.41	111.52	100	90.53	69.93	72.72	68.63	3.91
Marmoricola	0.00252	0.00596	124.47	100	92.53	103.21	90.9	106.99	100	109.25	87.03	91.23	83.24	4.19
Massilia	0.03106	0.04498	95.51	100	95.71	134.28	120.78	205.38	100	105.99	90.04	76.71	98.66	4.54
Mesorhizobium	0.00012	0.00066	54.3	100	153.25	113.28	148.25	115.46	100	133.25	96.3	94.78	85.39	4.18
Methylobacillus	0.00000	0.00003	5.72	100	110.77	111.84	51.47	110.47	100	29.39	48.75	29.87	45.88	3.5
Methylorosula	0.00028	0.00112	74.43	100	97.5	136.08	130.3	146.09	100	113.88	93.94	86.89	104.21	2.77
Methylotenera	0.00084	0.00272	25.65	100	105.02	103.85	85.19	113.75	100	33.21	46.06	29.81	34.99	3.65
Microbacterium	0.00113	0.00340	47.81	100	86.21	86.86	58.65	81.77	100	61.78	53.07	91.35	56.44	4.02
Microvirga	0.01117	0.01974	88.4	100	87.16	131.72	117.23	153.13	100	128.75	100.41	89.88	100.73	4.68
Mitsuaria	0.00000	0.00000	4.05	100	186.27	65.24	15.34	20.94	100	22.02	122.11	39.36	45.24	4.18
Mycobacterium	0.00003	0.00029	129.13	100	112.86	108.69	99.46	99.32	100	111.11	86.57	80.64	94.55	3.82
Niastella	0.00016	0.00078	42.54	100	122.65	99.72	100.77	100.19	100	84.83	103.93	84.33	76.76	3.08
Nitrospira	0.00452	0.00958	73.35	100	94.34	102.74	125.14	95.65	100	126.41	131.36	132.93	130.44	4.33
Nocardioides	0.00032	0.00122	116.55	100	104.1	103.25	81.1	96.92	100	74.47	77.12	105.21	88.06	4.35
Nodosilinea PCC 7104	0.00014	0.00072	4.44	100	365.77	164.16	285.09	537.94	100	62.96	108.1	61.08	85.74	3.94
Nonomuraea	0.02585	0.03826	164.23	100	135.86	128.69	121.44	149.45	100	83.14	112.95	136.41	120.34	3
Nordella	0.01871	0.03023	76.83	100	86.64	76.27	87.63	63.77	100	118.51	115.66	104.41	94.03	3.91
Ohtaekwangia	0.00106	0.00325	40.94	100	138.97	101.56	96.31	75.28	100	98.56	68.21	56.24	56.63	3.58
Oligoflexus	0.01147	0.02013	75.43	100	114.74	196.29	147.47	246.44	100	104.58	101.28	100.08	89.26	3.21
Paenarthrobacter	0.03072	0.04473	77.13	100	59	79.34	67.36	96.77	100	49.03	74	84.53	80.38	3.83
Paenisporosarcina	0.00772	0.01461	118.53	100	91.56	89.1	90.2	121.1	100	150.84	108.43	164.75	159.11	3.51
Parasegetibacter	0.00019	0.00085	48.8	100	106.53	143.79	108.75	176.22	100	88.84	104.44	88.17	81.87	3.45
Parviterribacter	0.01759	0.02895	114.51	100	108.64	114.32	95.35	119.53	100	103.39	84.25	100.1	82.02	3.03
Paucibacter	0.01434	0.02420	35.11	100	73.34	93.1	86.8	90.59	100	103.2	85.01	78.87	82.57	2.8
Pedobacter	0.00022	0.00093	60.67	100	87	121.29	111.08	157.41	100	98.06	91.41	94.85	118	3.81
Pedococcus Phycicoccus	0.00877	0.01637	117.36	100	104.5	101.22	71.6	82.76	100	67.41	65.76	114.7	94.73	2.75
Pedomicrobium	0.00069	0.00233	139.37	100	104.16	92.52	115.34	84.09	100	116.58	120.13	124.33	103.38	3.79
Pelobacter sp (uncultured)	0.01183	0.02049	81.57	100	83.24	99.92	87.49	48.4	100	152.75	158.34	87.91	71.97	2.92
Phenylobacterium	0.00460	0.00960	111.74	100	117.36	114.19	104.22	128.67	100	94.3	88.33	102.38	100.64	2.75
Pirellula	0.01454	0.02438	76.73	100	84.79	73.88	79.74	58.73	100	106.6	103.1	88.43	96.47	4.56
Planctomicrobium	0.00157	0.00442	53	100	82.99	69.53	75.57	46.5	100	81.54	74.74	57.08	75.76	2.97
Planctomycetales bacterium (uncultured)	0.00024	0.00099	52.01	100	86.92	90.13	109.59	63.5	100	104.4	86.69	67.59	90.35	2.96
Planctomycete (uncultured)	0.00003	0.00029	50.19	100	75.22	94.88	106.91	83.75	100	110.02	86.07	59.5	71.82	4.33
Polaromonas	0.00930	0.01714	55.72	100	93.34	74.33	101.92	80.41	100	92.42	100.91	91.08	78.08	2.77
Polyangium brachysporum group	0.00000	0.00002	37.24	100	86.63	121.47	76.64	82.31	100	114.09	52.41	57.47	63.86	3.66
Pontibacter	0.00940	0.01714	57.75	100	85.41	136.31	102.16	190.91	100	84.65	85.7	79.28	91.33	3.95
Promicromonospora	0.02417	0.03640	190.96	100	158.51	75.41	101.83	118.54	100	114.24	65.95	56.91	64.89	3.26
Pseudarthrobacter	0.00537	0.01086	84.97	100	77.45	138.88	100.57	146	100	69.17	89.54	103.35	95.64	3.1
Pseudoduganella	0.00011	0.00066	14.86	100	87.11	169.15	61.09	46.1	100	80.33	92.38	49.47	54.03	3.55
Pseudoflavitalea	0.00212	0.00527	63.89	100	109.19	79.98	183.11	73.52	100	315.91	176.06	114.63	117.23	2.97
Pseudolabrys	0.00509	0.01053	82.32	100	153.37	74.21	119.47	70.11	100	107.49	88.24	85.94	89.34	3.87
Pseudomuriella schumacherensis	0.00011	0.00066	9.04	100	92.62	107.42	123.27	137.2	100	162.94	194.47	120.17	126.2	3.48
Pseudorhodoplanes	0.00213	0.00527	55.69	100	112.11	103.87	145.19	94.01	100	149.76	130.24	114.67	105.78	3.02
Pseudoxanthomonas	0.00011	0.00066	39.43	100	179.05	92.26	68.45	79.34	100	72.05	68.2	52.08	38.59	3.99
Qipengyuania	0.00000	0.00004	33.59	100	86.64	113.9	64.24	93.25	100	64.59	66.28	76.4	74.62	3.83
Ramlibacter	0.00001	0.00009	55.16	100	88.81	130.57	121.36	152.85	100	102.93	82.29	53.2	58.2	4.08
Reyranella	0.00440	0.00940	78.84	100	108.23	89.66	118.3	76.81	100	119.41	121.64	85.51	89.13	3.35
Rhizobacter	0.00005	0.00038	57.28	100	112.86	111.25	109.72	125.42	100	89.86	80.62	73.57	67.43	3.96
Rhodobacter	0.00005	0.00036	45.12	100	154.54	188.95	178.91	210.64	100	60.03	66.67	65.67	53.72	3.52
Rhodocytophaga	0.02868	0.04199	52.13	100	87.56	109.5	97.61	132	100	97.81	121.12	110.42	114.44	3.21
Rhodopirellula	0.00004	0.00032	34.94	100	84.11	110.22	90.01	122.8	100	113.5	77.79	63.64	72.1	3.71
Rhodoplanes	0.04025	0.05585	80.4	100	93.11	72.59	87.46	58.75	100	103.24	116.88	100.44	93.22	3.69
Roseimicrobium	0.04645	0.06313	78.41	100	81.16	91.17	102.83	62.01	100	93.57	96.1	72.17	77.43	3.29
Roseomonas	0.00288	0.00658	65.46	100	101.84	141.54	139.67	208.01	100	110.7	95.93	90.18	80.99	3.18
Rubellimicrobium	0.00097	0.00307	55.45	100	85.14	144.84	128.94	194.48	100	88.45	86.67	88.12	78.28	4.16
Rubrobacter	0.00944	0.01714	107.74	100	106.81	123.91	112.37	132.26	100	109.31	107.23	107.84	123.59	4.52
Rubrobacterales bacterium (uncultured)	0.00000	0.00000	142.64	100	113.72	93.52	76.37	79.43	100	82.64	81.7	101.47	127.99	4.44
Rubrobacteria bacterium (uncultured)	0.00000	0.00002	133.1	100	123.22	104.38	81.68	98.33	100	82.09	88.35	110.12	125.76	3.93
Saccharothrix	0.03157	0.04547	56.66	100	74.64	78.66	78.51	113.29	100	66.31	70.17	60.58	81.93	3.17
Shimazuella	0.02083	0.03229	176.45	100	129.24	145.57	130.79	177.02	100	73.85	112.1	123.5	95.85	2.79
Shinella	0.00053	0.00185	17.43	100	133.61	80.34	54.36	78.55	100	56.69	77.75	62.36	46.07	3.6
Skermanella	0.03385	0.04797	106.51	100	109.91	136.22	161.04	188.5	100	98.68	101.87	121.24	112.65	4.41
Solirubrobacter	0.00005	0.00036	134.79	100	114.29	109.03	98.61	106.4	100	93.78	93.35	123.24	111.6	3.97
Sorangium	0.00215	0.00527	213.85	100	247.96	120.74	117.95	88.89	100	127.31	54.17	61.89	39.88	3.08
Sphingoaurantiacus	0.00024	0.00099	72.87	100	92.72	137.67	114.16	184.98	100	93.12	71.07	91.75	96.31	3.22
Sphingobium	0.00001	0.00010	6.51	100	117.69	148.59	78.58	126.93	100	50.67	51.95	47.45	40.18	4.7
Sphingomonas	0.00025	0.00103	88.32	100	102.32	113.86	84.73	109.96	100	97.32	78.45	85.9	95.57	4.65
Sphingopyxis	0.00012	0.00066	29.7	100	156.98	102.35	79.6	116.39	100	34.5	46.1	51.69	64.04	3.34
Steroidobacter	0.00032	0.00122	128.4	100	114.51	102.9	132.42	109.09	100	118.38	118.98	132.53	102.27	3.9
Streptomyces	0.00054	0.00185	69.59	100	102.58	75.42	79.73	81.61	100	87.19	71.23	81.07	81.15	4.2
Sumerlaea	0.00106	0.00325	49.38	100	76.33	105.62	104.13	102.95	100	130.93	122.19	109.87	85.69	3.09
Synechococcus IR11	0.00187	0.00502	4.86	100	426.01	340.41	446.08	642.72	100	295.95	105.58	96.44	140.27	3.55
Tahibacter	0.00672	0.01300	30.74	100	76.94	61.78	46.78	52.77	100	112.37	52.53	41.82	53.16	3.09
Tepidisphaera	0.00308	0.00697	51.92	100	68.48	115.57	117.08	97.03	100	108.41	102.77	54.86	64.63	3.19
Terrimonas	0.00310	0.00697	57.62	100	93.9	92.63	102.48	76.39	100	123.05	103.4	72.42	70.95	3.85
Thermomicrobia bacterium (uncultured)	0.04136	0.05709	108.08	100	113.5	93.8	105.86	123.29	100	77.72	78.57	81.08	105.2	2.63
Truepera	0.00000	0.00002	40.65	100	68.9	103.99	123.09	128.05	100	139.03	119.11	138.52	88.47	3.55
Tumebacillus	0.00190	0.00502	205.98	100	195.02	112.13	135.76	178.05	100	79.54	126.8	284.58	190.6	3.71
Tychonema CCAP 1459 11B	0.00668	0.01300	27.04	100	90.7	64.94	118.9	107.72	100	66.79	176.66	122.13	107.17	4.09
Variovorax	0.00010	0.00062	60.83	100	113.44	76.83	79.79	84.4	100	68.31	73.19	61.59	44.08	3.81
Yonghaparkia	0.00000	0.00002	11.37	100	96.24	129.17	65.29	127.42	100	63.24	68.06	83.94	91.99	3.71

**Table 7:** Data grouped by label. Percentual standardization of values vs. CRD_t1 or CRD_t2. Negative differences greater than 10% are highlighted in red, while positive differences greater than 10% are highlighted in green.

Genus	*p*-Value	FDR	t0	CFD_t1	CRD_t1	MYC_t1	PSBA_t1	PSBA + MYC_t1	CFD_t2	CRD_t2	MYC_t2	PSBA_t2	PSBA + MYC_t2	LDA Score
Acidibacter	0.00015	0.00072	62.8	97.71	100	97.01	142.88	92.48	76.04	100	66.44	59.42	55.48	3.96
Acidimicrobiia bacterium (uncultured)	0.00002	0.00018	147.81	79.47	100	78.12	74.35	83.68	116.1	100	107.5	135.58	127.4	4.14
Acidobacteria bacterium (uncultured)	0.02573	0.03826	119.44	116.53	100	94.79	121.61	81.63	90.36	100	110.6	103.88	88.71	4.4
Acidobacteriaceae bacterium (uncultured)	0.00440	0.00940	118.11	113.33	100	94.58	121.34	90.02	87.81	100	104.44	106.06	92.45	4.53
Acidobacteriales bacterium (uncultured)	0.00047	0.00167	154.84	108.39	100	90.96	115.3	81.76	81.35	100	109.96	123.24	97.48	4.39
Acidovorax	0.00000	0.00007	7.1	113.61	100	224.58	74.67	47.99	237.91	100	107.47	176.94	144.27	3.38
Acinetobacter	0.00585	0.01175	2.14	64.35	100	1.3	1.44	17.46	135.34	100	171.19	54.2	61.31	4.94
Actinobacterium (uncultured)	0.00137	0.00398	111.79	82.73	100	79.06	64.21	66.19	98.71	100	95.42	104.07	104.32	4.05
Actinomadura	0.01713	0.02837	120.82	98.02	100	103.8	101.85	108.49	94.75	100	83.71	73.47	99.66	2.8
Actinomycetales bacterium (uncultured)	0.00000	0.00005	87.3	84.93	100	85.12	66.49	83.88	122.14	100	105.88	137.57	170.94	3.46
Adhaeribacter	0.00047	0.00167	87.04	111.25	100	154.51	144.04	181.2	95.78	100	101.81	81.57	100.39	3.91
Aeromicrobium	0.00028	0.00112	49.77	107.37	100	133.16	100.34	106.29	119.49	100	73.64	45.13	47.3	4.61
Allorhizobium Neorhizobium Pararhizobium Rhizobium	0.00216	0.00527	54.88	91.72	100	97.27	79.92	103.54	111.93	100	94.64	87.77	79.68	4.5
Alsobacter	0.01771	0.02897	93.43	96.86	100	158.66	145.69	187.36	81.93	100	93.37	93.65	95.52	3.11
Altererythrobacter	0.00032	0.00122	19.44	56.31	100	53.86	57.98	52.49	103.73	100	72.18	72.02	68.2	3.65
Amaricoccus	0.03566	0.05000	76.4	81.34	100	110.99	112.09	144.19	107.23	100	98.67	133.77	132.47	3.2
Aminobacter	0.00000	0.00000	71.59	141.35	100	795.33	760.01	808.64	79.92	100	141.44	144.55	132.33	3.53
Ammoniphilus	0.01802	0.02930	100.1	71.83	100	83.4	86.64	105.89	89.53	100	67.56	95.45	63.11	3.02
Amycolatopsis	0.00000	0.00000	38.17	141.49	100	100.82	30.17	61.68	332.06	100	141.75	122.35	218.25	3.71
Anaerolinea	0.02455	0.03675	328.02	111.93	100	126.57	120.62	136.83	130.99	100	137.94	165.48	229.24	3.61
Anaeromyxobacter	0.00231	0.00557	173.8	98.91	100	83.29	111.7	112.45	105.88	100	129.22	187.09	129.17	3.63
Arenimonas	0.00005	0.00036	43.15	114.34	100	103.88	93.36	97.43	137.67	100	55.64	58.83	55.32	3.29
Aridibacter	0.00931	0.01714	74.49	128.69	100	125.11	119.01	143.77	101.41	100	83.93	78.04	110.28	3.35
Armatimonadetes bacterium (uncultured)	0.00015	0.00072	127.55	103.67	100	112.49	111.51	126.23	85.01	100	97.45	100.63	110.12	3.03
Arthrobacter	0.00662	0.01299	110.97	122.23	100	145.32	132.79	171.31	115.77	100	120.23	134.28	139.16	5.28
Azohydromonas	0.00000	0.00001	67.56	105.75	100	120.95	104.68	150.13	99.41	100	83.41	75.04	59.26	3.54
Blastocatella	0.00834	0.01568	70.31	116.42	100	123.31	99.51	116.14	107.47	100	105.39	99.76	112.57	3.5
Blastococcus	0.00613	0.01222	93.55	101.48	100	128.33	114.82	152.01	91.3	100	86.52	93.39	92.48	4.28
Blastopirellula	0.00173	0.00478	108.99	104.94	100	91.76	122.11	86.64	85.37	100	119.37	121.37	95.98	3.05
Bosea	0.00116	0.00347	50.97	94.47	100	119.6	106.69	149.48	92.68	100	87.18	97.58	62.15	3.7
Bradyrhizobium	0.00046	0.00167	64.87	84.01	100	76.81	108.63	80.21	94.36	100	99.03	85.96	77.23	3.95
Burkholderia Caballeronia Paraburkholderia	0.01243	0.02138	165.35	76.78	100	90.49	76.26	71.31	109.07	100	50.18	45.48	43.19	3.54
Caenimonas	0.00005	0.00038	68.88	109.53	100	140.98	136.24	168.41	105.2	100	78.61	62.23	64.34	4.13
Candidatus Udaeobacter	0.02707	0.03985	110.45	105.43	100	64.8	72	45.85	86.72	100	100.13	93.4	97.14	4.15
Candidatus Xiphinematobacter	0.00981	0.01769	136.81	124.97	100	99.35	120	88.89	83.82	100	110.85	105.7	103.84	4.66
Caulobacter	0.03307	0.04737	33.19	36.97	100	31.2	27.59	33.86	117.27	100	86.05	63.01	50.64	3.31
Cellulomonas	0.00188	0.00502	112.75	75.06	100	113.35	115.82	177.48	102.04	100	106.84	174.56	100.79	2.98
Chloroflexi bacterium (uncultured)	0.00000	0.00003	106.14	92.26	100	105.3	93.64	102.55	113.11	100	103.39	112.59	132.08	4.32
Chloroflexus sp (uncultured)	0.00001	0.00010	97.61	99.27	100	80.27	57.87	60.95	120.63	100	88.41	72.24	106.15	3.84
Chthoniobacter	0.00083	0.00270	80.22	124.86	100	130.99	148.39	134.8	88.4	100	93.55	69.58	82.19	4.28
Clostridium sensu stricto 1	0.01543	0.02572	156.51	87.22	100	75.28	88.33	97.18	146	100	197.57	167.09	157.99	3.25
Cohnella	0.00242	0.00577	126.36	81.09	100	92.34	104.3	122.71	81.2	100	96.51	157.04	141.99	3.44
Comamonas	0.00001	0.00010	50.01	99.78	100	101.8	110.54	94.55	115.8	100	89.06	91.9	62.94	3.18
Conexibacter	0.00396	0.00882	85.37	80.44	100	101.96	77.27	90.32	113.33	100	92.6	120.94	113.08	2.86
Conexibacter sp (uncultured)	0.00001	0.00009	126.52	86.58	100	94.13	86.95	96.15	113.58	100	111.69	140.18	151.98	2.98
Cupriavidus	0.00012	0.00067	65.94	94.69	100	68.9	84.52	124.97	47.52	100	56.51	50.6	47.89	3.53
Desulfuromonadales bacterium (uncultured)	0.03380	0.04797	167.71	110.29	100	102.54	161.05	128.58	102.61	100	103.42	141.48	139.67	3.17
Devosia	0.00001	0.00011	31.3	70.8	100	70.55	84.91	66.03	108.67	100	78.25	80.6	68.53	4.34
Domibacillus	0.01899	0.03050	126.77	114.98	100	88.22	102.77	137.53	123.8	100	117.38	120.94	181.78	3.37
Dongia	0.00020	0.00090	184.46	130.49	100	89.8	100.94	81.81	81.36	100	98.37	86.55	84.51	3.83
Dyadobacter	0.00168	0.00469	44.07	102.51	100	86.4	51.83	71.15	291.76	100	241.18	105.88	97.42	3.82
Ensifer	0.00003	0.00029	68.97	106.61	100	102.34	94.22	114.62	95.42	100	108.45	90.79	92.72	3.92
Enterobacter	0.00412	0.00903	150.52	765.15	100	103.27	56.3	161.41	587.02	100	111.41	266.57	100.22	4.28
Ferruginibacter	0.02223	0.03386	84.79	111.17	100	129.05	128.21	134.5	94.45	100	102.12	84.96	91.53	3.33
Fictibacillus	0.00007	0.00049	162.4	92.57	100	98.18	87.32	119.23	49.96	100	49.73	82.97	84.19	3.82
Fimbriiglobus	0.00217	0.00527	81.06	107.46	100	89.92	116.28	90.81	89.9	100	107.24	104.28	104.46	3.8
Flavisolibacter	0.00142	0.00405	76.18	115.73	100	138.77	124.31	136.44	88.75	100	79.91	65.47	74.38	3.84
Flavitalea	0.00002	0.00017	61.01	110.9	100	142.68	131.13	123.68	82.75	100	88.94	75.07	74.07	3.3
Gaiella	0.00000	0.00001	122.81	84.01	100	92.14	76.12	85.4	120.9	100	106.67	137.11	146.64	5.06
Gaiella sp (uncultured)	0.00000	0.00007	113.95	88.66	100	110.68	74.69	104.59	124.04	100	116.6	148.8	154.29	3.99
Gemmata	0.01289	0.02204	93.09	108.77	100	80.94	98.19	77.46	89.41	100	100.61	95.29	102.02	4.41
Gemmatimonadalesbacterium (uncultured)	0.00264	0.00620	141.46	115.99	100	72.43	95.38	58.67	86.84	100	101.88	110.22	107.89	3.46
Gemmatimonadetesbacterium (uncultured)	0.03426	0.04829	108.35	116.83	100	125.64	154.69	97.2	78.27	100	91.11	69.93	63.66	3.47
Gemmatimonas	0.00012	0.00066	74.49	124.74	100	131.39	139.65	115.99	91.86	100	94.15	73.67	73.19	3.52
Haliangium	0.01926	0.03075	66.24	94.1	100	118.21	106.44	66.61	91.69	100	92.88	50.7	60.19	3.66
Herbinix	0.01033	0.01837	143.92	86.02	100	77.94	84.76	109.11	100.48	100	81.75	153.76	124.68	2.88
Herpetosiphon	0.02142	0.03281	58.65	89.74	100	126.96	81.74	111.17	142.11	100	92.58	93.83	94.73	3.88
Hirschia	0.00001	0.00009	19.03	65.61	100	77.82	129.9	79.55	30.32	100	34.44	17.05	14.66	3.82
Hyphomicrobium	0.01002	0.01794	139.51	102.08	100	93.1	94.2	82.2	109.93	100	121.94	137.41	124.1	3.04
Iamia	0.03915	0.05460	93.63	89.24	100	88.5	71.48	67.73	128.16	100	84.42	86.22	89.05	3.64
Iamia sp (uncultured)	0.00142	0.00405	66.64	107.71	100	135.76	96.61	103.38	177.43	100	99.85	85.37	88.02	2.93
Ideonella	0.00401	0.00884	10.88	38.82	100	42.72	33.77	45.99	82.38	100	97.61	47.68	57.06	3.16
Ilumatobacter	0.04702	0.06357	100.44	78.89	100	70.48	55.97	60.76	149.42	100	117.81	136.16	156.78	3.47
Knoellia	0.00217	0.00527	114.47	103.4	100	110.56	103.85	126.46	114.61	100	98.75	133.74	114.09	4.4
Kribbella	0.00081	0.00269	119.46	84.95	100	84.66	78.65	85.11	119.71	100	106.01	113.59	119.53	3.05
Lacibacter	0.01180	0.02049	49.95	122.39	100	126.01	92.79	93.58	138.89	100	93.63	64.01	64.47	3.24
Lechevalieria	0.00000	0.00004	52.56	91.1	100	63.11	50.02	59.65	157.09	100	84.73	90.99	92.35	3.98
Leptolyngbya EcFYyyy 00	0.00001	0.00015	1.95	45.17	100	183.17	129.56	146.44	74.58	100	214.75	62.31	56.64	4.59
Litorilinea	0.04367	0.05996	79.01	95.72	100	107.92	119.76	131.82	110.08	100	108.14	95.05	121.57	3.4
Luedemannella	0.00007	0.00049	166.82	66.71	100	78.66	85.55	100.98	126.09	100	126.57	195.9	145.98	3.45
Luteitalea	0.00522	0.01073	80.17	95.05	100	112.9	123.93	134.65	91.48	100	97.14	133.96	89.81	3.42
Luteolibacter	0.00005	0.00037	35.13	67.08	100	71.69	73.92	66.99	122.4	100	94.67	71.2	70.03	3.71
Lutispora sp (uncultured)	0.00432	0.00939	319.31	104.16	100	133.81	130.28	129.75	118.88	100	150.14	214.92	193.66	3.64
Lysobacter	0.00455	0.00958	77.69	105.18	100	125.01	91.94	117.29	110.46	100	77.25	80.32	75.81	3.91
Marmoricola	0.00252	0.00596	134.53	108.08	100	111.54	98.25	115.63	91.53	100	79.66	83.51	76.19	4.19
Massilia	0.03106	0.04498	99.79	104.48	100	140.29	126.19	214.58	94.35	100	84.95	72.37	93.09	4.54
Mesorhizobium	0.00012	0.00066	35.43	65.25	100	73.92	96.74	75.34	75.05	100	72.27	71.13	64.08	4.18
Methylobacillus	0.00000	0.00003	5.16	90.27	100	100.97	46.46	99.73	340.26	100	165.87	101.63	156.12	3.5
Methylorosula	0.00028	0.00112	76.34	102.56	100	139.56	133.64	149.84	87.81	100	82.5	76.3	91.52	2.77
Methylotenera	0.00084	0.00272	24.43	95.22	100	98.88	81.12	108.31	301.13	100	138.69	89.76	105.38	3.65
Microbacterium	0.00113	0.00340	55.45	115.99	100	100.75	68.03	94.84	161.87	100	85.9	147.87	91.36	4.02
Microvirga	0.01117	0.01974	101.43	114.73	100	151.12	134.5	175.69	77.67	100	77.99	69.81	78.23	4.68
Mitsuaria	0.00000	0.00000	2.17	53.69	100	35.03	8.24	11.24	454.18	100	554.6	178.77	205.47	4.18
Mycobacterium	0.00003	0.00029	114.42	88.61	100	96.31	88.13	88	90	100	77.91	72.58	85.1	3.82
Niastella	0.00016	0.00078	34.69	81.53	100	81.3	82.17	81.69	117.88	100	122.51	99.41	90.49	3.08
Nitrospira	0.00452	0.00958	77.75	106	100	108.9	132.65	101.38	79.11	100	103.91	105.16	103.19	4.33
Nocardioides	0.00032	0.00122	111.96	96.06	100	99.18	77.9	93.11	134.28	100	103.56	141.28	118.24	4.35
Nodosilinea PCC 7104	0.00014	0.00072	1.21	27.34	100	44.88	77.94	147.07	158.82	100	171.68	97.01	136.17	3.94
Nonomuraea	0.02585	0.03826	120.89	73.61	100	94.72	89.38	110.01	120.28	100	135.85	164.07	144.75	3
Nordella	0.01871	0.03023	88.67	115.42	100	88.03	101.14	73.6	84.38	100	97.59	88.1	79.34	3.91
Ohtaekwangia	0.00106	0.00325	29.46	71.96	100	73.08	69.3	54.17	101.46	100	69.21	57.06	57.46	3.58
Oligoflexus	0.01147	0.02013	65.74	87.15	100	171.08	128.53	214.79	95.62	100	96.84	95.69	85.35	3.21
Paenarthrobacter	0.03072	0.04473	130.73	169.49	100	134.48	114.18	164.02	203.95	100	150.92	172.4	163.93	3.83
Paenisporosarcina	0.00772	0.01461	129.46	109.22	100	97.31	98.52	132.27	66.29	100	71.88	109.22	105.48	3.51
Parasegetibacter	0.00019	0.00085	45.81	93.87	100	134.98	102.09	165.42	112.56	100	117.55	99.24	92.15	3.45
Parviterribacter	0.01759	0.02895	105.41	92.05	100	105.23	87.77	110.02	96.72	100	81.49	96.82	79.33	3.03
Paucibacter	0.01434	0.02420	47.88	136.36	100	126.95	118.36	123.52	96.9	100	82.38	76.43	80.01	2.8
Pedobacter	0.00022	0.00093	69.74	114.94	100	139.41	127.68	180.92	101.98	100	93.22	96.73	120.34	3.81
Pedococcus Phycicoccus	0.00877	0.01637	112.31	95.7	100	96.86	68.52	79.2	148.35	100	97.55	170.15	140.53	2.75
Pedomicrobium	0.00069	0.00233	133.81	96.01	100	88.83	110.74	80.74	85.78	100	103.05	106.64	88.67	3.79
Pelobacter sp (uncultured)	0.01183	0.02049	97.99	120.13	100	120.03	105.1	58.14	65.47	100	103.66	57.55	47.12	2.92
Phenylobacterium	0.00460	0.00960	95.21	85.21	100	97.3	88.8	109.63	106.04	100	93.67	108.57	106.72	2.75
Pirellula	0.01454	0.02438	90.49	117.94	100	87.13	94.04	69.26	93.81	100	96.72	82.95	90.5	4.56
Planctomicrobium	0.00157	0.00442	63.86	120.49	100	83.78	91.06	56.02	122.64	100	91.67	70	92.91	2.97
Planctomycetales bacterium (uncultured)	0.00024	0.00099	59.83	115.05	100	103.7	126.08	73.06	95.79	100	83.04	64.74	86.54	2.96
Planctomycete (uncultured)	0.00003	0.00029	66.73	132.95	100	126.15	142.15	111.35	90.89	100	78.24	54.09	65.28	4.33
Polaromonas	0.00930	0.01714	59.7	107.13	100	79.63	109.19	86.14	108.2	100	109.18	98.55	84.48	2.77
Polyangium brachysporum group	0.00000	0.00002	42.99	115.43	100	140.21	88.46	95.01	87.65	100	45.94	50.37	55.97	3.66
Pontibacter	0.00940	0.01714	67.62	117.08	100	159.6	119.62	223.52	118.13	100	101.24	93.65	107.89	3.95
Promicromonospora	0.02417	0.03640	120.47	63.09	100	47.58	64.24	74.79	87.54	100	57.73	49.82	56.8	3.26
Pseudarthrobacter	0.00537	0.01086	109.71	129.12	100	179.32	129.85	188.51	144.57	100	129.45	149.41	138.27	3.1
Pseudoduganella	0.00011	0.00066	17.06	114.8	100	194.18	70.13	52.92	124.48	100	114.99	61.59	67.26	3.55
Pseudoflavitalea	0.00212	0.00527	58.51	91.58	100	73.25	167.7	67.34	31.65	100	55.73	36.29	37.11	2.97
Pseudolabrys	0.00509	0.01053	53.67	65.2	100	48.39	77.9	45.72	93.04	100	82.1	79.96	83.12	3.87
Pseudomuriella schumacherensis	0.00011	0.00066	9.76	107.97	100	115.99	133.1	148.14	61.37	100	119.35	73.75	77.45	3.48
Pseudorhodoplanes	0.00213	0.00527	49.67	89.2	100	92.65	129.5	83.85	66.77	100	86.97	76.57	70.63	3.02
Pseudoxanthomonas	0.00011	0.00066	22.02	55.85	100	51.53	38.23	44.31	138.8	100	94.66	72.29	53.56	3.99
Qipengyuania	0.00000	0.00004	38.76	115.42	100	131.47	74.14	107.63	154.83	100	102.63	118.29	115.54	3.83
Ramlibacter	0.00001	0.00009	62.11	112.6	100	147.02	136.65	172.1	97.16	100	79.95	51.68	56.55	4.08
Reyranella	0.00440	0.00940	72.85	92.39	100	82.84	109.3	70.97	83.74	100	101.86	71.61	74.64	3.35
Rhizobacter	0.00005	0.00038	50.76	88.61	100	98.57	97.22	111.13	111.29	100	89.72	81.87	75.04	3.96
Rhodobacter	0.00005	0.00036	29.2	64.71	100	122.26	115.77	136.3	166.58	100	111.05	109.39	89.49	3.52
Rhodocytophaga	0.02868	0.04199	59.54	114.21	100	125.07	111.49	150.76	102.24	100	123.84	112.9	117.01	3.21
Rhodopirellula	0.00004	0.00032	41.55	118.9	100	131.05	107.02	146	88.1	100	68.54	56.07	63.52	3.71
Rhodoplanes	0.04025	0.05585	86.35	107.4	100	77.97	93.94	63.1	96.86	100	113.22	97.29	90.3	3.69
Roseimicrobium	0.04645	0.06313	96.61	123.21	100	112.32	126.69	76.4	106.87	100	102.7	77.13	82.75	3.29
Roseomonas	0.00288	0.00658	64.27	98.19	100	138.98	137.15	204.25	90.33	100	86.66	81.46	73.16	3.18
Rubellimicrobium	0.00097	0.00307	65.13	117.45	100	170.12	151.44	228.41	113.06	100	97.98	99.63	88.5	4.16
Rubrobacter	0.00944	0.01714	100.87	93.62	100	116.01	105.2	123.83	91.48	100	98.1	98.65	113.06	4.52
Rubrobacterales bacterium (uncultured)	0.00000	0.00000	125.43	87.94	100	82.24	67.16	69.84	121.01	100	98.86	122.78	154.88	4.44
Rubrobacteria bacterium (uncultured)	0.00000	0.00002	108.02	81.15	100	84.71	66.29	79.79	121.82	100	107.63	134.16	153.21	3.93
Saccharothrix	0.03157	0.04547	75.91	133.98	100	105.39	105.19	151.79	150.8	100	105.82	91.36	123.54	3.17
Shimazuella	0.02083	0.03229	136.53	77.37	100	112.63	101.2	136.97	135.41	100	151.8	167.24	129.8	2.79
Shinella	0.00053	0.00185	13.05	74.85	100	60.13	40.69	58.79	176.38	100	137.13	109.99	81.26	3.6
Skermanella	0.03385	0.04797	96.91	90.98	100	123.94	146.52	171.5	101.33	100	103.23	122.86	114.15	4.41
Solirubrobacter	0.00005	0.00036	117.94	87.5	100	95.4	86.28	93.1	106.64	100	99.55	131.42	119.01	3.97
Sorangium	0.00215	0.00527	86.25	40.33	100	48.69	47.57	35.85	78.55	100	42.55	48.61	31.32	3.08
Sphingoaurantiacus	0.00024	0.00099	78.59	107.85	100	148.48	123.13	199.51	107.38	100	76.32	98.52	103.43	3.22
Sphingobium	0.00001	0.00010	5.53	84.97	100	126.26	66.77	107.85	197.37	100	102.54	93.65	79.3	4.7
Sphingomonas	0.00025	0.00103	86.32	97.74	100	111.29	82.81	107.47	102.75	100	80.61	88.26	98.2	4.65
Sphingopyxis	0.00012	0.00066	18.92	63.7	100	65.2	50.71	74.14	289.82	100	133.61	149.8	185.6	3.34
Steroidobacter	0.00032	0.00122	112.12	87.33	100	89.86	115.64	95.27	84.48	100	100.51	111.95	86.39	3.9
Streptomyces	0.00054	0.00185	67.84	97.49	100	73.52	77.73	79.56	114.7	100	81.7	92.98	93.07	4.2
Sumerlaea	0.00106	0.00325	64.69	131.01	100	138.36	136.41	134.87	76.38	100	93.33	83.92	65.45	3.09
Synechococcus IR11	0.00187	0.00502	1.14	23.47	100	79.9	104.71	150.87	33.79	100	35.68	32.59	47.4	3.55
Tahibacter	0.00672	0.01300	39.95	129.97	100	80.3	60.81	68.59	88.99	100	46.75	37.21	47.31	3.09
Tepidisphaera	0.00308	0.00697	75.82	146.03	100	168.77	170.98	141.7	92.25	100	94.8	50.6	59.62	3.19
Terrimonas	0.00310	0.00697	61.36	106.49	100	98.64	109.13	81.35	81.27	100	84.03	58.85	57.66	3.85
Thermomicrobia bacterium (uncultured)	0.04136	0.05709	95.22	88.1	100	82.64	93.26	108.62	128.68	100	101.1	104.33	135.36	2.63
Truepera	0.00000	0.00002	59	145.14	100	150.94	178.65	185.85	71.93	100	85.67	99.63	63.63	3.55
Tumebacillus	0.00190	0.00502	105.62	51.28	100	57.5	69.61	91.3	125.72	100	159.41	357.77	239.62	3.71
Tychonema CCAP 1459 11B	0.00668	0.01300	29.82	110.25	100	71.6	131.09	118.76	149.73	100	264.51	182.86	160.47	4.09
Variovorax	0.00010	0.00062	53.62	88.15	100	67.73	70.33	74.4	146.4	100	107.14	90.17	64.53	3.81
Yonghaparkia	0.00000	0.00002	11.81	103.9	100	134.21	67.84	132.39	158.14	100	107.62	132.74	145.48	3.71

**Table 8:** Data grouped by treatment. Percentual standardization of values vs. t0. Negative differences greater than 10% are highlighted in red, while positive differences greater than 10% are highlighted in green.

Genus	*p*-Value	FDR	t0	CFD	CRD	MYC	PSBA	PSBA + MYC	LDA Score
Acidibacter	0.00026	0.00196	100	177.39	210.58	164.9	191.57	146.41	3.79
Acidimicrobiia bacterium (uncultured)	0.00034	0.00241	100	50.7	54.34	48.18	52.96	54.71	4.09
Acidobacteriales bacterium (uncultured)	0.01264	0.02937	100	69.88	75.16	77.7	90.07	66.27	4.19
Acidovorax	0.00006	0.00104	100	1347.41	934.23	1739.6	932.94	670.62	3.11
Actinobacterium (uncultured)	0.00567	0.01549	100	65.65	73.75	62.54	58.92	59.79	4.02
Aeromicrobium	0.00018	0.00155	100	267.48	234.05	229.79	161.1	175.42	4.49
Allorhizobium Neorhizobium Pararhizobium Rhizobium	0.00113	0.00474	100	207.37	201.71	194.37	169.89	183.24	4.36
Alsobacter	0.01434	0.03257	100	110.04	124.56	150.01	144.5	172.18	2.97
Altererythrobacter	0.00014	0.00146	100	290.37	397.53	237.33	250.19	235.61	3.51
Amaricoccus	0.00851	0.02128	100	113.56	121.7	127	148.61	171.37	3.1
Aminobacter	0.00000	0.00000	100	184.31	176.93	680	685.6	759.35	3.34
Amycolatopsis	0.00103	0.00456	100	1435.17	507.39	692.37	500.04	809.66	3.45
Anaerolinea	0.01738	0.03871	100	28.79	24.19	31.18	33.2	41.42	3.58
Anaeromyxobacter	0.00021	0.00165	100	51.91	50.93	52.91	73.59	61.44	3.58
Arenimonas	0.00057	0.00307	100	273.32	218.17	173.06	168.36	176.53	3.27
Aridibacter	0.01313	0.03025	100	159.82	139.54	143.2	136.39	178.44	3.27
Armatimonadetes bacterium (uncultured)	0.01982	0.04340	100	67.92	71.3	74.52	76.01	86.59	2.87
Arthrobacter	0.00067	0.00325	100	117.82	99.26	130.62	132.62	152.83	5.2
Azohydromonas	0.00193	0.00683	100	240.03	236.72	228.32	199.56	209.37	3.34
Blastocatella	0.00530	0.01498	100	149.57	133.26	151.71	132.76	154.13	3.35
Bosea	0.00107	0.00466	100	207.49	222	224.71	225.56	232.34	3.54
Bradyrhizobium	0.00047	0.00273	100	141.63	158.55	141.31	153.76	124.6	3.89
Burkholderia Caballeronia Paraburkholderia	0.00876	0.02171	100	58.97	63.02	43.08	37.97	36.65	3.48
Caenimonas	0.00377	0.01188	100	183.98	171.9	178.79	160.7	193.43	3.94
Cellulomonas	0.04245	0.08045	100	66.13	76.53	83.6	107.54	116.93	2.71
Chloroflexi bacterium (uncultured)	0.00771	0.01984	100	79.71	79.16	81.64	80.19	91.38	4.09
Chloroflexus sp (uncultured)	0.00001	0.00045	100	95.31	88.08	73.13	56.27	69.35	3.79
Chthonomonas	0.02807	0.05767	100	130.07	130.32	120.33	131.04	150.69	2.53
Clostridium sensu stricto 1	0.01438	0.03257	100	50.13	47.2	54.59	53.71	56.02	3.13
Clostridium sensu stricto 8	0.02672	0.05533	100	53.39	58.16	56.83	67.68	67.99	2.78
Cohnella	0.00127	0.00509	100	53.5	65.94	61.25	82.68	87.39	3.35
Comamonas	0.00042	0.00272	100	311.03	282.43	268.31	278.19	206.81	3
Conexibacter sp (uncultured)	0.00047	0.00273	100	58.06	60.52	59.73	63.8	70.67	2.84
Cupriavidus	0.01615	0.03628	100	171.75	286.17	175.54	170.52	194.75	3.29
Defluviicoccus	0.01052	0.02557	100	73.67	70.43	75.95	98.44	107.26	2.72
Desmochloris halophila	0.00010	0.00136	100	3389.26	2325.21	2344.77	3150.88	1823.76	3.3
Desulfuromonadales bacterium (uncultured)	0.00284	0.00970	100	66.53	62.6	64.7	94.4	83.2	3.14
Devosia	0.00002	0.00064	100	230.58	267.85	195.41	222.77	183.48	4.23
Dongia	0.00144	0.00529	100	65.62	64.28	61.73	59.54	52.44	3.76
Dyadobacter	0.00067	0.00325	100	415.05	215.86	354.92	167.22	178.1	3.62
Ensifer	0.00133	0.00518	100	177.81	177.84	191.1	163.95	178.95	3.77
Ferrimicrobium sp (uncultured)	0.03729	0.07311	100	79.64	79.49	81.54	86.81	67.03	2.91
Fictibacillus	0.00011	0.00136	100	46.45	66.72	47.27	56.69	67.76	3.74
Flavitalea	0.00003	0.00067	100	199.98	213.8	234.21	206.44	199.48	3.21
Gaiella	0.00065	0.00325	100	59.73	61.82	59.03	59.93	66.2	4.91
Gaiella sp (uncultured)	0.00782	0.01994	100	67.95	67.3	74.46	67.62	83.25	3.78
Gemmatimonadales bacterium (uncultured)	0.04590	0.08447	100	73.72	73.02	64.83	75.24	58.89	3.31
Gemmatimonas	0.00017	0.00147	100	171.4	162.56	178.15	164.04	148.71	3.46
Herbidospora	0.04913	0.08761	100	84.66	143.88	89.56	151.54	132.11	2.75
Herbinix	0.00344	0.01114	100	52.32	57.07	44.74	63.77	67	2.82
Hirschia	0.00028	0.00207	100	359.23	878.92	417.22	446.36	314.2	3.65
Iamia	0.01109	0.02647	100	101.36	95.31	81.85	74.31	73.34	3.52
Ideonella	0.00021	0.00165	100	418.12	750.61	486.18	293.94	383.1	3.06
Knoellia	0.03739	0.07311	100	85.69	79.04	82.32	92.66	97.45	4.11
Kribbella	0.00435	0.01326	100	77.6	76.97	72.78	72.81	76.81	2.95
Lacibacter	0.00211	0.00736	100	260.94	199.77	217.27	156.68	161.61	3.2
Lechevalieria	0.00046	0.00273	100	252.6	200.75	151.51	143.7	149.2	3.79
Leptolyngbya EcFYyyy 00	0.00002	0.00064	100	2140.01	3878.75	7397.56	4138.1	4870.86	4.48
Litorilinea	0.03752	0.07311	100	121.46	118.59	127.53	128.35	152.68	3.29
Luedemannella	0.00007	0.00108	100	39.5	45.44	42.89	55.95	53.81	3.39
Luteitalea	0.00135	0.00518	100	125.26	134.49	140.46	173.91	151.16	3.32
Luteolibacter	0.00039	0.00258	100	205.42	232.16	185.95	169.16	162.31	3.56
Lutispora sp (uncultured)	0.00067	0.00325	100	27.9	25.41	35.17	41.36	39.38	3.61
Marmoricola	0.00515	0.01487	100	77.75	78.22	73.58	70.8	75.72	4.08
Mesorhizobium	0.00002	0.00067	100	195.82	279.35	203.92	234.84	197.12	4.17
metagenome	0.00406	0.01251	100	78.96	83.84	81.02	82.88	79.27	4.28
Methylobacillus	0.00011	0.00136	100	6346.25	2576.58	3757.78	2084.31	3283.36	3.26
Methylotenera	0.00009	0.00136	100	1027.66	481.25	598.02	414.26	504.4	3.42
Microbacterium	0.00372	0.01186	100	317.9	221.95	205.53	256.2	201.55	3.84
Microlunatus	0.04250	0.08045	100	88.37	91.93	97.19	106.18	117.57	3.74
Microvirga	0.04487	0.08314	100	121.42	132.82	139	124.61	154.6	4.55
Mitsuaria	0.00001	0.00045	100	8902	3989.43	10737.74	3207.06	3325.77	3.94
Mycobacterium	0.00012	0.00136	100	77.18	86.43	74.79	69.53	75.09	3.72
Nakamurella	0.04455	0.08314	100	106.58	111.13	124.89	130.79	164.98	2.53
Niastella	0.00023	0.00182	100	302.84	301.36	314.82	274.72	256.95	2.96
Nitrosospira	0.04718	0.08622	100	128.25	153.5	165.62	188.07	251.72	3.01
Nitrospira	0.00112	0.00474	100	128.76	140.9	150.26	165.85	142.5	4.3
Nocardia	0.02207	0.04605	100	173.32	232.51	171.13	141.82	179.46	2.87
Nocardioides	0.04227	0.08045	100	81.16	73.15	72.81	75.04	76.25	4.15
Nodosilinea PCC 7104	0.00001	0.00046	100	4351.84	6152.39	5445.13	5182.46	9239.08	3.82
Nonomuraea	0.02141	0.04575	100	58.94	65.05	70.9	75.85	81.19	2.89
Nordella	0.02831	0.05771	100	124.19	126.44	119.25	118.75	95.32	3.65
Ohtaekwangia	0.00037	0.00258	100	257.81	303.46	214.48	193.93	170.66	3.51
Paenisporosarcina	0.00535	0.01498	100	66.41	75.17	63.1	77.97	91.2	3.35
Parasegetibacter	0.00067	0.00325	100	200.35	196.12	246.56	197.73	273.25	3.27
Paucibacter	0.02168	0.04597	100	251.29	216.81	222.48	209.49	223.8	2.71
Pelobacter sp (uncultured)	0.03482	0.06990	100	124.15	147.03	163.32	108.89	72.96	2.73
Planctomicrobium	0.02204	0.04605	100	226.42	185.98	166.52	146.69	136.9	2.82
Planctomycetales bacterium (uncultured)	0.00765	0.01984	100	239.03	232.73	213.01	201.93	181.64	2.81
planctomycete (uncultured)	0.00016	0.00147	100	212.45	199.06	191.81	173.65	164.77	4.21
Polyangium brachysporum group	0.00006	0.00104	100	444.11	469.82	325.42	280.97	297.46	3.44
Promicromonospora	0.00656	0.01757	100	70.61	92.25	49.68	51.94	60.14	3.17
Pseudarthrobacter	0.00094	0.00421	100	135.08	98.31	149.09	137.97	160.45	2.99
Pseudoduganella	0.00000	0.00045	100	753.66	628.22	942.24	411.93	371.72	3.46
Pseudoflavitalea	0.01219	0.02860	100	124.3	230.9	144.89	196.08	111.96	2.79
Pseudomuriella schumacherensis	0.00013	0.00138	100	827.59	959.36	1123.69	1011.69	1156.88	3.35
Pseudorhodoplanes	0.00011	0.00136	100	168.97	219.25	197.05	221.16	168.25	2.89
Pseudoxanthomonas	0.00003	0.00067	100	406.76	428.75	312.86	232.6	207.73	3.85
Qipengyuania	0.01122	0.02654	100	239.22	187.33	222.14	164.66	215.17	3.59
Ramlibacter	0.00010	0.00136	100	223.56	217.31	227.14	180.7	223.55	3.94
Reyranella	0.01046	0.02557	100	131.96	150.49	142	133.64	108.26	3.21
Rhizobacter	0.00015	0.00146	100	198.81	198.73	186.53	177.83	188.96	3.87
Rhodocytophaga	0.00182	0.00653	100	194.31	180.21	225.14	202.27	240.95	3.18
Rhodopirellula	0.00006	0.00104	100	283.52	279.76	263.72	218.17	286.28	3.58
Roseimicrobium	0.03548	0.07069	100	129.74	113.48	121.88	113.18	89.18	3.18
Roseomonas	0.02030	0.04374	100	156.66	166.66	183.05	179.08	235.63	2.97
Rubellimicrobium	0.00140	0.00529	100	183.98	159.75	208.63	198.94	261.53	3.97
Rubrobacterales bacterium (uncultured)	0.00053	0.00299	100	58.77	59.46	51.26	50.83	57.88	4.34
Rubrobacteria bacterium (uncultured)	0.00345	0.01114	100	64.84	68.67	62.29	60.71	71.56	3.78
Shimazuella	0.02890	0.05845	100	63.81	62.82	80.92	80.87	86.18	2.68
Shinella	0.00003	0.00067	100	648.01	588.03	514.6	381.15	399.09	3.52
Skermanella	0.04926	0.08761	100	96.41	100.41	113.44	135.57	148.3	4.21
Solirubrobacter	0.02018	0.04374	100	65.59	69.12	66.12	71.7	72.23	3.84
Sorangium	0.00618	0.01672	100	87.82	140.01	63.58	67.45	45.86	2.97
Sphingobium	0.00001	0.00045	100	2823.28	1945.12	2204.04	1578.67	1819.13	4.53
Sphingopyxis	0.00457	0.01377	100	333.74	321.33	242.15	219.49	313.11	3.07
Streptomyces	0.00012	0.00136	100	156.52	147.53	114.91	125.93	126.09	4.11
Sumerlaea	0.00135	0.00518	100	178.91	178.96	201.03	190.76	175.5	3.03
Synechococcus IR11	0.00015	0.00146	100	2697.85	9323.56	5150.12	6201.85	9492.62	3.41
Tahibacter	0.00313	0.01051	100	391.14	381.88	221.81	171.65	202.85	2.94
Terrimonas	0.00067	0.00325	100	183.68	200.71	181.88	159.1	134.73	3.71
Truepera	0.00123	0.00503	100	472.17	570.19	563.01	635.07	447.48	3.34
Tumebacillus	0.00814	0.02054	100	42.44	61.79	49.98	84.66	78.93	3.6
Variovorax	0.00038	0.00258	100	223.94	190.06	169.6	152.89	132.72	3.64
Yonghaparkia	0.00682	0.01806	100	587.2	516.48	637.19	410.86	749.05	3.5

**Table 9:** Data grouped by treatment. Percentual standardization of values vs. CFD. Negative differences greater than 10% are highlighted in red, while positive differences greater than 10% are highlighted in green.

Genus	*p*-Value	FDR	t0	CFD	CRD	MYC	PSBA	PSBA + MYC	LDA Score
Acidibacter	0.00026	0.00196	56.37	100	118.71	92.96	107.99	82.54	3.79
Acidimicrobiia bacterium (uncultured)	0.00034	0.00241	197.24	100	107.18	95.04	104.46	107.92	4.09
Acidobacteriales bacterium (uncultured)	0.01264	0.02937	143.11	100	107.57	111.19	128.9	94.84	4.19
Acidovorax	0.00006	0.00104	7.42	100	69.34	129.11	69.24	49.77	3.11
Actinobacterium (uncultured)	0.00567	0.01549	152.33	100	112.34	95.27	89.76	91.09	4.02
Aeromicrobium	0.00018	0.00155	37.39	100	87.5	85.91	60.23	65.58	4.49
Allorhizobium Neorhizobium Pararhizobium Rhizobium	0.00113	0.00474	48.22	100	97.27	93.73	81.93	88.36	4.36
Alsobacter	0.01434	0.03257	90.88	100	113.2	136.32	131.32	156.47	2.97
Altererythrobacter	0.00014	0.00146	34.44	100	136.91	81.74	86.16	81.14	3.51
Amaricoccus	0.00851	0.02128	88.06	100	107.17	111.84	130.87	150.91	3.1
Aminobacter	0.00000	0.00000	54.26	100	96	368.95	371.99	412	3.34
Amycolatopsis	0.00103	0.00456	6.97	100	35.35	48.24	34.84	56.42	3.45
Anaerolinea	0.01738	0.03871	347.38	100	84.05	108.3	115.32	143.88	3.58
Anaeromyxobacter	0.00021	0.00165	192.62	100	98.1	101.91	141.74	118.35	3.58
Arenimonas	0.00057	0.00307	36.59	100	79.82	63.32	61.6	64.59	3.27
Aridibacter	0.01313	0.03025	62.57	100	87.31	89.6	85.34	111.65	3.27
Armatimonadetes bacterium (uncultured)	0.01982	0.04340	147.22	100	104.97	109.71	111.91	127.49	2.87
Arthrobacter	0.00067	0.00325	84.87	100	84.25	110.86	112.56	129.71	5.2
Azohydromonas	0.00193	0.00683	41.66	100	98.62	95.12	83.14	87.23	3.34
Blastocatella	0.00530	0.01498	66.86	100	89.09	101.43	88.76	103.05	3.35
Bosea	0.00107	0.00466	48.2	100	106.99	108.3	108.71	111.98	3.54
Bradyrhizobium	0.00047	0.00273	70.61	100	111.95	99.78	108.56	87.98	3.89
Burkholderia Caballeronia Paraburkholderia	0.00876	0.02171	169.59	100	106.87	73.07	64.39	62.15	3.48
Caenimonas	0.00377	0.01188	54.35	100	93.43	97.18	87.34	105.13	3.94
Cellulomonas	0.04245	0.08045	151.22	100	115.73	126.41	162.62	176.81	2.71
Chloroflexi bacterium (uncultured)	0.00771	0.01984	125.46	100	99.31	102.43	100.61	114.65	4.09
Chloroflexus sp (uncultured)	0.00001	0.00045	104.92	100	92.41	76.73	59.04	72.77	3.79
Chthonomonas	0.02807	0.05767	76.88	100	100.19	92.51	100.74	115.85	2.53
Clostridium sensu stricto 1	0.01438	0.03257	199.47	100	94.15	108.89	107.13	111.74	3.13
Clostridium sensu stricto 8	0.02672	0.05533	187.32	100	108.95	106.46	126.78	127.36	2.78
Cohnella	0.00127	0.00509	186.91	100	123.25	114.49	154.54	163.34	3.35
Comamonas	0.00042	0.00272	32.15	100	90.8	86.27	89.44	66.49	3
Conexibacter sp (uncultured)	0.00047	0.00273	172.23	100	104.23	102.87	109.87	121.71	2.84
Cupriavidus	0.01615	0.03628	58.23	100	166.62	102.21	99.28	113.39	3.29
Defluviicoccus	0.01052	0.02557	135.73	100	95.59	103.09	133.61	145.59	2.72
Desmochloris halophila	0.00010	0.00136	2.95	100	68.61	69.18	92.97	53.81	3.3
Desulfuromonadales bacterium (uncultured)	0.00284	0.00970	150.31	100	94.1	97.25	141.9	125.06	3.14
Devosia	0.00002	0.00064	43.37	100	116.16	84.75	96.61	79.58	4.23
Dongia	0.00144	0.00529	152.4	100	97.97	94.07	90.74	79.92	3.76
Dyadobacter	0.00067	0.00325	24.09	100	52.01	85.51	40.29	42.91	3.62
Ensifer	0.00133	0.00518	56.24	100	100.02	107.48	92.2	100.64	3.77
Ferrimicrobium sp (uncultured)	0.03729	0.07311	125.56	100	99.81	102.37	109	84.16	2.91
Fictibacillus	0.00011	0.00136	215.28	100	143.63	101.77	122.05	145.88	3.74
Flavitalea	0.00003	0.00067	50	100	106.91	117.12	103.23	99.75	3.21
Gaiella	0.00065	0.00325	167.43	100	103.51	98.84	100.35	110.84	4.91
Gaiella sp (uncultured)	0.00782	0.01994	147.16	100	99.04	109.57	99.51	122.51	3.78
Gemmatimonadales bacterium (uncultured)	0.04590	0.08447	135.66	100	99.06	87.95	102.07	79.89	3.31
Gemmatimonas	0.00017	0.00147	58.34	100	94.84	103.94	95.71	86.76	3.46
Herbidospora	0.04913	0.08761	118.11	100	169.95	105.78	178.99	156.05	2.75
Herbinix	0.00344	0.01114	191.15	100	109.08	85.52	121.9	128.06	2.82
Hirschia	0.00028	0.00207	27.84	100	244.67	116.14	124.26	87.46	3.65
Iamia	0.01109	0.02647	98.66	100	94.03	80.75	73.31	72.36	3.52
Ideonella	0.00021	0.00165	23.92	100	179.52	116.28	70.3	91.63	3.06
Knoellia	0.03739	0.07311	116.7	100	92.24	96.06	108.13	113.72	4.11
Kribbella	0.00435	0.01326	128.87	100	99.2	93.79	93.83	98.98	2.95
Lacibacter	0.00211	0.00736	38.32	100	76.56	83.26	60.05	61.93	3.2
Lechevalieria	0.00046	0.00273	39.59	100	79.48	59.98	56.89	59.06	3.79
Leptolyngbya EcFYyyy 00	0.00002	0.00064	4.67	100	181.25	345.68	193.37	227.61	4.48
Litorilinea	0.03752	0.07311	82.33	100	97.64	105	105.67	125.7	3.29
Luedemannella	0.00007	0.00108	253.14	100	115.04	108.58	141.63	136.22	3.39
Luteitalea	0.00135	0.00518	79.83	100	107.37	112.13	138.84	120.68	3.32
Luteolibacter	0.00039	0.00258	48.68	100	113.02	90.52	82.35	79.01	3.56
Lutispora sp (uncultured)	0.00067	0.00325	358.38	100	91.07	126.06	148.23	141.14	3.61
Marmoricola	0.00515	0.01487	128.62	100	100.61	94.63	91.06	97.39	4.08
Mesorhizobium	0.00002	0.00067	51.07	100	142.66	104.14	119.92	100.66	4.17
metagenome	0.00406	0.01251	126.64	100	106.18	102.6	104.97	100.4	4.28
Methylobacillus	0.00011	0.00136	1.58	100	40.6	59.21	32.84	51.74	3.26
Methylotenera	0.00009	0.00136	9.73	100	46.83	58.19	40.31	49.08	3.42
Microbacterium	0.00372	0.01186	31.46	100	69.82	64.65	80.59	63.4	3.84
Microlunatus	0.04250	0.08045	113.16	100	104.03	109.98	120.16	133.05	3.74
Microvirga	0.04487	0.08314	82.36	100	109.38	114.48	102.62	127.32	4.55
Mitsuaria	0.00001	0.00045	1.12	100	44.81	120.62	36.03	37.36	3.94
Mycobacterium	0.00012	0.00136	129.56	100	111.99	96.9	90.08	97.28	3.72
Nakamurella	0.04455	0.08314	93.82	100	104.26	117.18	122.71	154.79	2.53
Niastella	0.00023	0.00182	33.02	100	99.51	103.95	90.71	84.85	2.96
Nitrosospira	0.04718	0.08622	77.97	100	119.68	129.14	146.64	196.27	3.01
Nitrospira	0.00112	0.00474	77.66	100	109.43	116.7	128.8	110.67	4.3
Nocardia	0.02207	0.04605	57.7	100	134.15	98.74	81.83	103.54	2.87
Nocardioides	0.04227	0.08045	123.22	100	90.13	89.72	92.46	93.96	4.15
Nodosilinea PCC 7104	0.00001	0.00046	2.3	100	141.37	125.12	119.09	212.3	3.82
Nonomuraea	0.02141	0.04575	169.66	100	110.37	120.29	128.68	137.75	2.89
Nordella	0.02831	0.05771	80.52	100	101.81	96.02	95.62	76.75	3.65
Ohtaekwangia	0.00037	0.00258	38.79	100	117.71	83.19	75.22	66.2	3.51
Paenisporosarcina	0.00535	0.01498	150.58	100	113.19	95.02	117.4	137.32	3.35
Parasegetibacter	0.00067	0.00325	49.91	100	97.89	123.06	98.69	136.38	3.27
Paucibacter	0.02168	0.04597	39.8	100	86.28	88.54	83.37	89.06	2.71
Pelobacter sp (uncultured)	0.03482	0.06990	80.54	100	118.43	131.55	87.71	58.76	2.73
Planctomicrobium	0.02204	0.04605	44.17	100	82.14	73.55	64.79	60.46	2.82
Planctomycetales bacterium (uncultured)	0.00765	0.01984	41.84	100	97.37	89.11	84.48	75.99	2.81
planctomycete (uncultured)	0.00016	0.00147	47.07	100	93.7	90.29	81.74	77.56	4.21
Polyangium brachysporum group	0.00006	0.00104	22.52	100	105.79	73.28	63.27	66.98	3.44
Promicromonospora	0.00656	0.01757	141.62	100	130.66	70.36	73.57	85.18	3.17
Pseudarthrobacter	0.00094	0.00421	74.03	100	72.78	110.37	102.13	118.78	2.99
Pseudoduganella	0.00000	0.00045	13.27	100	83.36	125.02	54.66	49.32	3.46
Pseudoflavitalea	0.01219	0.02860	80.45	100	185.76	116.56	157.75	90.08	2.79
Pseudomuriella schumacherensis	0.00013	0.00138	12.08	100	115.92	135.78	122.24	139.79	3.35
Pseudorhodoplanes	0.00011	0.00136	59.18	100	129.76	116.62	130.89	99.57	2.89
Pseudoxanthomonas	0.00003	0.00067	24.58	100	105.41	76.92	57.18	51.07	3.85
Qipengyuania	0.01122	0.02654	41.8	100	78.31	92.86	68.83	89.95	3.59
Ramlibacter	0.00010	0.00136	44.73	100	97.21	101.6	80.83	100	3.94
Reyranella	0.01046	0.02557	75.78	100	114.04	107.61	101.27	82.04	3.21
Rhizobacter	0.00015	0.00146	50.3	100	99.96	93.82	89.44	95.04	3.87
Rhodocytophaga	0.00182	0.00653	51.47	100	92.75	115.87	104.1	124.01	3.18
Rhodopirellula	0.00006	0.00104	35.27	100	98.67	93.02	76.95	100.97	3.58
Roseimicrobium	0.03548	0.07069	77.08	100	87.47	93.95	87.24	68.74	3.18
Roseomonas	0.02030	0.04374	63.83	100	106.38	116.85	114.31	150.41	2.97
Rubellimicrobium	0.00140	0.00529	54.35	100	86.83	113.4	108.13	142.15	3.97
Rubrobacterales bacterium (uncultured)	0.00053	0.00299	170.17	100	101.18	87.23	86.5	98.49	4.34
Rubrobacteria bacterium (uncultured)	0.00345	0.01114	154.24	100	105.92	96.08	93.64	110.38	3.78
Shimazuella	0.02890	0.05845	156.72	100	98.45	126.81	126.74	135.07	2.68
Shinella	0.00003	0.00067	15.43	100	90.74	79.41	58.82	61.59	3.52
Skermanella	0.04926	0.08761	103.72	100	104.15	117.66	140.62	153.82	4.21
Solirubrobacter	0.02018	0.04374	152.45	100	105.37	100.81	109.31	110.12	3.84
Sorangium	0.00618	0.01672	113.87	100	159.43	72.4	76.81	52.23	2.97
Sphingobium	0.00001	0.00045	3.54	100	68.9	78.07	55.92	64.43	4.53
Sphingopyxis	0.00457	0.01377	29.96	100	96.28	72.55	65.76	93.82	3.07
Streptomyces	0.00012	0.00136	63.89	100	94.25	73.41	80.46	80.56	4.11
Sumerlaea	0.00135	0.00518	55.89	100	100.03	112.36	106.62	98.09	3.03
Synechococcus IR11	0.00015	0.00146	3.71	100	345.59	190.9	229.88	351.86	3.41
Tahibacter	0.00313	0.01051	25.57	100	97.63	56.71	43.88	51.86	2.94
Terrimonas	0.00067	0.00325	54.44	100	109.27	99.02	86.62	73.35	3.71
Truepera	0.00123	0.00503	21.18	100	120.76	119.24	134.5	94.77	3.34
Tumebacillus	0.00814	0.02054	235.61	100	145.58	117.75	199.46	185.96	3.6
Variovorax	0.00038	0.00258	44.65	100	84.87	75.73	68.27	59.26	3.64
Yonghaparkia	0.00682	0.01806	17.03	100	87.96	108.51	69.97	127.56	3.5

**Table 10:** Data grouped by treatment. Percentual standardization of values vs. CRD. Negative differences greater than 10% are highlighted in red, while positive differences greater than 10% are highligthed in green.

Genus	*p*-Value	FDR	t0	CFD	CRD	MYC	PSBA	PSBA + MYC	LDA Score
Acidibacter	0.00026	0.00196	47.49	84.24	100	78.31	90.97	69.53	3.79
Acidimicrobiia bacterium (uncultured)	0.00034	0.00241	184.03	93.3	100	88.67	97.47	100.69	4.09
Acidobacteriales bacterium (uncultured)	0.01264	0.02937	133.04	92.97	100	103.37	119.83	88.16	4.19
Acidovorax	0.00006	0.00104	10.7	144.23	100	186.21	99.86	71.78	3.11
Actinobacterium (uncultured)	0.00567	0.01549	135.6	89.02	100	84.81	79.9	81.08	4.02
Aeromicrobium	0.00018	0.00155	42.73	114.29	100	98.18	68.83	74.95	4.49
Allorhizobium Neorhizobium Pararhizobium Rhizobium	0.00113	0.00474	49.58	102.8	100	96.36	84.22	90.84	4.36
Alsobacter	0.01434	0.03257	80.28	88.34	100	120.43	116.01	138.23	2.97
Altererythrobacter	0.00014	0.00146	25.16	73.04	100	59.7	62.94	59.27	3.51
Amaricoccus	0.00851	0.02128	82.17	93.31	100	104.36	122.11	140.81	3.1
Aminobacter	0.00000	0.00000	56.52	104.17	100	384.33	387.49	429.17	3.34
Amycolatopsis	0.00103	0.00456	19.71	282.86	100	136.46	98.55	159.57	3.45
Anaerolinea	0.01738	0.03871	413.32	118.98	100	128.86	137.22	171.19	3.58
Anaeromyxobacter	0.00021	0.00165	196.36	101.94	100	103.89	144.5	120.64	3.58
Arenimonas	0.00057	0.00307	45.84	125.28	100	79.32	77.17	80.92	3.27
Aridibacter	0.01313	0.03025	71.67	114.53	100	102.63	97.75	127.88	3.27
Armatimonadetes bacterium (uncultured)	0.01982	0.04340	140.26	95.27	100	104.52	106.61	121.45	2.87
Arthrobacter	0.00067	0.00325	100.74	118.7	100	131.59	133.61	153.97	5.2
Azohydromonas	0.00193	0.00683	42.24	101.4	100	96.45	84.3	88.45	3.34
Blastocatella	0.00530	0.01498	75.04	112.25	100	113.85	99.63	115.67	3.35
Bosea	0.00107	0.00466	45.05	93.46	100	101.22	101.6	104.66	3.54
Bradyrhizobium	0.00047	0.00273	63.07	89.33	100	89.13	96.98	78.59	3.89
Burkholderia Caballeronia Paraburkholderia	0.00876	0.02171	158.69	93.57	100	68.37	60.25	58.15	3.48
Caenimonas	0.00377	0.01188	58.17	107.03	100	104.01	93.48	112.53	3.94
Cellulomonas	0.04245	0.08045	130.66	86.41	100	109.23	140.52	152.78	2.71
Chloroflexi bacterium (uncultured)	0.00771	0.01984	126.33	100.7	100	103.14	101.31	115.45	4.09
Chloroflexus sp (uncultured)	0.00001	0.00045	113.54	108.21	100	83.03	63.88	78.74	3.79
Chthonomonas	0.02807	0.05767	76.73	99.81	100	92.33	100.55	115.63	2.53
Clostridium sensu stricto 1	0.01438	0.03257	211.86	106.21	100	115.65	113.78	118.68	3.13
Clostridium sensu stricto 8	0.02672	0.05533	171.93	91.79	100	97.72	116.37	116.9	2.78
Cohnella	0.00127	0.00509	151.65	81.14	100	92.89	125.39	132.53	3.35
Comamonas	0.00042	0.00272	35.41	110.13	100	95	98.5	73.23	3
Conexibacter sp (uncultured)	0.00047	0.00273	165.24	95.95	100	98.7	105.42	116.78	2.84
Cupriavidus	0.01615	0.03628	34.94	60.02	100	61.34	59.59	68.05	3.29
Defluviicoccus	0.01052	0.02557	141.99	104.61	100	107.84	139.77	152.29	2.72
Desmochloris halophila	0.00010	0.00136	4.3	145.76	100	100.84	135.51	78.43	3.3
Desulfuromonadales bacterium (uncultured)	0.00284	0.00970	159.74	106.27	100	103.35	150.8	132.9	3.14
Devosia	0.00002	0.00064	37.33	86.08	100	72.95	83.17	68.5	4.23
Dongia	0.00144	0.00529	155.56	102.08	100	96.03	92.62	81.58	3.76
Dyadobacter	0.00067	0.00325	46.33	192.28	100	164.42	77.47	82.51	3.62
Ensifer	0.00133	0.00518	56.23	99.98	100	107.46	92.19	100.62	3.77
Ferrimicrobium sp (uncultured)	0.03729	0.07311	125.8	100.19	100	102.57	109.2	84.32	2.91
Fictibacillus	0.00011	0.00136	149.89	69.62	100	70.86	84.97	101.57	3.74
Flavitalea	0.00003	0.00067	46.77	93.54	100	109.55	96.56	93.3	3.21
Gaiella	0.00065	0.00325	161.75	96.61	100	95.49	96.94	107.08	4.91
Gaiella sp (uncultured)	0.00782	0.01994	148.59	100.97	100	110.63	100.48	123.7	3.78
Gemmatimonadales bacterium (uncultured)	0.04590	0.08447	136.95	100.95	100	88.79	103.04	80.65	3.31
Gemmatimonas	0.00017	0.00147	61.52	105.44	100	109.59	100.91	91.48	3.46
Herbidospora	0.04913	0.08761	69.5	58.84	100	62.24	105.32	91.82	2.75
Herbinix	0.00344	0.01114	175.23	91.67	100	78.4	111.75	117.4	2.82
Hirschia	0.00028	0.00207	11.38	40.87	100	47.47	50.79	35.75	3.65
Iamia	0.01109	0.02647	104.92	106.35	100	85.88	77.96	76.95	3.52
Ideonella	0.00021	0.00165	13.32	55.7	100	64.77	39.16	51.04	3.06
Knoellia	0.03739	0.07311	126.51	108.41	100	104.15	117.23	123.29	4.11
Kribbella	0.00435	0.01326	129.91	100.81	100	94.55	94.59	99.78	2.95
Lacibacter	0.00211	0.00736	50.06	130.62	100	108.76	78.43	80.89	3.2
Lechevalieria	0.00046	0.00273	49.81	125.82	100	75.47	71.58	74.32	3.79
Leptolyngbya EcFYyyy 00	0.00002	0.00064	2.58	55.17	100	190.72	106.69	125.58	4.48
Litorilinea	0.03752	0.07311	84.33	102.42	100	107.54	108.23	128.75	3.29
Luedemannella	0.00007	0.00108	220.05	86.93	100	94.38	123.12	118.41	3.39
Luteitalea	0.00135	0.00518	74.35	93.13	100	104.43	129.31	112.39	3.32
Luteolibacter	0.00039	0.00258	43.07	88.48	100	80.09	72.86	69.91	3.56
Lutispora sp (uncultured)	0.00067	0.00325	393.53	109.81	100	138.42	162.76	154.98	3.61
Marmoricola	0.00515	0.01487	127.84	99.39	100	94.06	90.51	96.8	4.08
Mesorhizobium	0.00002	0.00067	35.8	70.1	100	73	84.06	70.56	4.17
metagenome	0.00406	0.01251	119.27	94.18	100	96.63	98.86	94.55	4.28
Methylobacillus	0.00011	0.00136	3.88	246.31	100	145.84	80.89	127.43	3.26
Methylotenera	0.00009	0.00136	20.78	213.54	100	124.26	86.08	104.81	3.42
Microbacterium	0.00372	0.01186	45.06	143.23	100	92.6	115.43	90.81	3.84
Microlunatus	0.04250	0.08045	108.78	96.12	100	105.72	115.5	127.89	3.74
Microvirga	0.04487	0.08314	75.29	91.42	100	104.66	93.82	116.4	4.55
Mitsuaria	0.00001	0.00045	2.51	223.14	100	269.15	80.39	83.36	3.94
Mycobacterium	0.00012	0.00136	115.7	89.3	100	86.53	80.44	86.87	3.72
Nakamurella	0.04455	0.08314	89.99	95.91	100	112.39	117.69	148.46	2.53
Niastella	0.00023	0.00182	33.18	100.49	100	104.47	91.16	85.26	2.96
Nitrosospira	0.04718	0.08622	65.15	83.55	100	107.9	122.53	163.99	3.01
Nitrospira	0.00112	0.00474	70.97	91.38	100	106.64	117.7	101.14	4.3
Nocardia	0.02207	0.04605	43.01	74.54	100	73.6	61	77.19	2.87
Nocardioides	0.04227	0.08045	136.71	110.95	100	99.54	102.59	104.24	4.15
Nodosilinea PCC 7104	0.00001	0.00046	1.63	70.73	100	88.5	84.23	150.17	3.82
Nonomuraea	0.02141	0.04575	153.72	90.61	100	108.99	116.59	124.81	2.89
Nordella	0.02831	0.05771	79.09	98.22	100	94.32	93.92	75.39	3.65
Ohtaekwangia	0.00037	0.00258	32.95	84.95	100	70.68	63.9	56.24	3.51
Paenisporosarcina	0.00535	0.01498	133.03	88.35	100	83.95	103.72	121.32	3.35
Parasegetibacter	0.00067	0.00325	50.99	102.16	100	125.72	100.82	139.33	3.27
Paucibacter	0.02168	0.04597	46.12	115.9	100	102.61	96.62	103.22	2.71
Pelobacter sp (uncultured)	0.03482	0.06990	68.01	84.44	100	111.08	74.06	49.62	2.73
Planctomicrobium	0.02204	0.04605	53.77	121.74	100	89.54	78.87	73.61	2.82
Planctomycetales bacterium (uncultured)	0.00765	0.01984	42.97	102.71	100	91.52	86.76	78.05	2.81
planctomycete (uncultured)	0.00016	0.00147	50.24	106.73	100	96.36	87.23	82.77	4.21
Polyangium brachysporum group	0.00006	0.00104	21.28	94.53	100	69.26	59.8	63.31	3.44
Promicromonospora	0.00656	0.01757	108.4	76.54	100	53.85	56.31	65.19	3.17
Pseudarthrobacter	0.00094	0.00421	101.72	137.41	100	151.66	140.34	163.22	2.99
Pseudoduganella	0.00000	0.00045	15.92	119.97	100	149.98	65.57	59.17	3.46
Pseudoflavitalea	0.01219	0.02860	43.31	53.83	100	62.75	84.92	48.49	2.79
Pseudomuriella schumacherensis	0.00013	0.00138	10.42	86.26	100	117.13	105.45	120.59	3.35
Pseudorhodoplanes	0.00011	0.00136	45.61	77.07	100	89.87	100.87	76.74	2.89
Pseudoxanthomonas	0.00003	0.00067	23.32	94.87	100	72.97	54.25	48.45	3.85
Qipengyuania	0.01122	0.02654	53.38	127.7	100	118.58	87.89	114.86	3.59
Ramlibacter	0.00010	0.00136	46.02	102.88	100	104.52	83.15	102.87	3.94
Reyranella	0.01046	0.02557	66.45	87.69	100	94.36	88.8	71.94	3.21
Rhizobacter	0.00015	0.00146	50.32	100.04	100	93.86	89.48	95.08	3.87
Rhodocytophaga	0.00182	0.00653	55.49	107.82	100	124.93	112.24	133.7	3.18
Rhodopirellula	0.00006	0.00104	35.75	101.35	100	94.27	77.99	102.33	3.58
Roseimicrobium	0.03548	0.07069	88.12	114.32	100	107.4	99.73	78.58	3.18
Roseomonas	0.02030	0.04374	60	94	100	109.84	107.45	141.39	2.97
Rubellimicrobium	0.00140	0.00529	62.6	115.16	100	130.59	124.53	163.71	3.97
Rubrobacterales bacterium (uncultured)	0.00053	0.00299	168.19	98.83	100	86.21	85.49	97.34	4.34
Rubrobacteria bacterium (uncultured)	0.00345	0.01114	145.62	94.41	100	90.71	88.41	104.21	3.78
Shimazuella	0.02890	0.05845	159.19	101.58	100	128.81	128.74	137.2	2.68
Shinella	0.00003	0.00067	17.01	110.2	100	87.51	64.82	67.87	3.52
Skermanella	0.04926	0.08761	99.59	96.01	100	112.97	135.01	147.69	4.21
Solirubrobacter	0.02018	0.04374	144.68	94.9	100	95.67	103.74	104.5	3.84
Sorangium	0.00618	0.01672	71.42	62.72	100	45.41	48.18	32.76	2.97
Sphingobium	0.00001	0.00045	5.14	145.15	100	113.31	81.16	93.52	4.53
Sphingopyxis	0.00457	0.01377	31.12	103.86	100	75.36	68.31	97.44	3.07
Streptomyces	0.00012	0.00136	67.78	106.1	100	77.89	85.36	85.47	4.11
Sumerlaea	0.00135	0.00518	55.88	99.97	100	112.33	106.59	98.06	3.03
Synechococcus IR11	0.00015	0.00146	1.07	28.94	100	55.24	66.52	101.81	3.41
Tahibacter	0.00313	0.01051	26.19	102.42	100	58.08	44.95	53.12	2.94
Terrimonas	0.00067	0.00325	49.82	91.51	100	90.62	79.27	67.13	3.71
Truepera	0.00123	0.00503	17.54	82.81	100	98.74	111.38	78.48	3.34
Tumebacillus	0.00814	0.02054	161.84	68.69	100	80.88	137.01	127.73	3.6
Variovorax	0.00038	0.00258	52.61	117.83	100	89.24	80.44	69.83	3.64
Yonghaparkia	0.00682	0.01806	19.36	113.69	100	123.37	79.55	145.03	3.5

## Data Availability

The genomic sequences were included in the BioProject PRJNA916628, titled “Impact of PGPB bacteria and AM fungi inocula on resident communities associated with tomato roots”, available in NCBI database https://submit.ncbi.nlm.nih.gov/subs/sra/SUB12451409/overview, accessed on 20 December 2022.

## Supplementary Materials

The following supporting information can be downloaded at: https://www.mdpi.com/article/10.3390/microorganisms11081891/s1, Figure S1. Weather data from Landlab weather station recorded during 2022.
