# Supplementary figures and images for: PGPB and/or AM Fungi Consortia Affect Tomato Native Rhizosphere Microbiota
Source: Microorganisms. 2023 Jul 26;11(8):1891. doi: 10.3390/microorganisms11081891 (PMC10458106; doi:10.3390/microorganisms11081891)

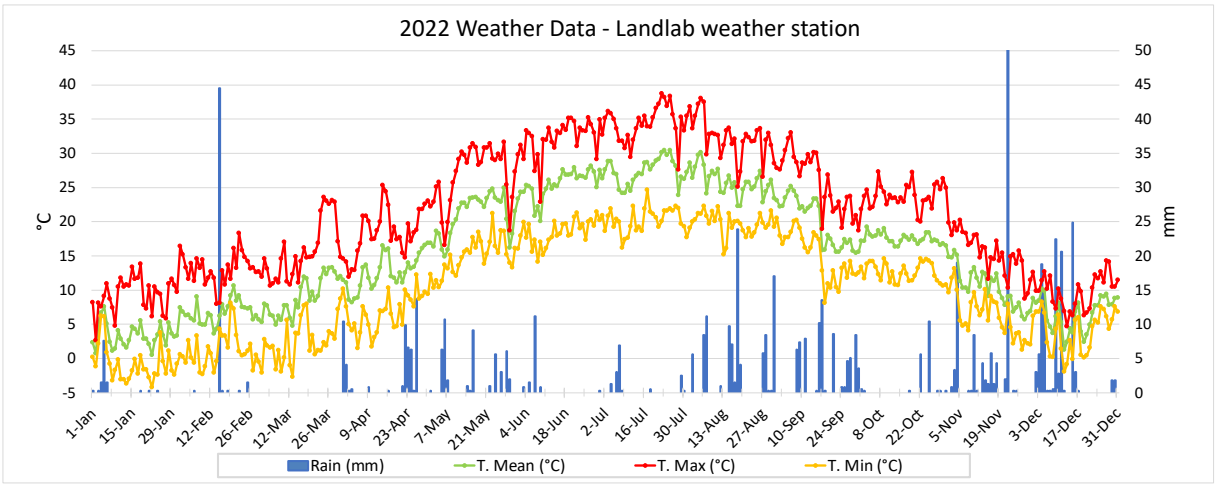

Figure S1. Weather data from Landlab weather station recorded during 2022.

Supplement: Supplementary file 1 [file microorganisms-11-01891-s001.zip › microorganisms-2506334-supplementary.pdf]
